# Supplementary material for: An improved pathway for autonomous bioluminescence imaging in eukaryotes
Source: Nat Methods. 2024 Jan 22;21(3):406–10. doi: 10.1038/s41592-023-02152-y (PMC10927554; doi:10.1038/s41592-023-02152-y)
Supplement: Supplementary file 1 — Supplementary Figs. 1–26 and Tables 1–5. [file 41592_2023_2152_MOESM1_ESM.pdf]

nature methods

Brief Communication https://doi.org/10.1038/s41592-023-02152-y

An improved pathway for autonomous bioluminescence imaging in eukaryotes

In the format provided by the authors and unedited

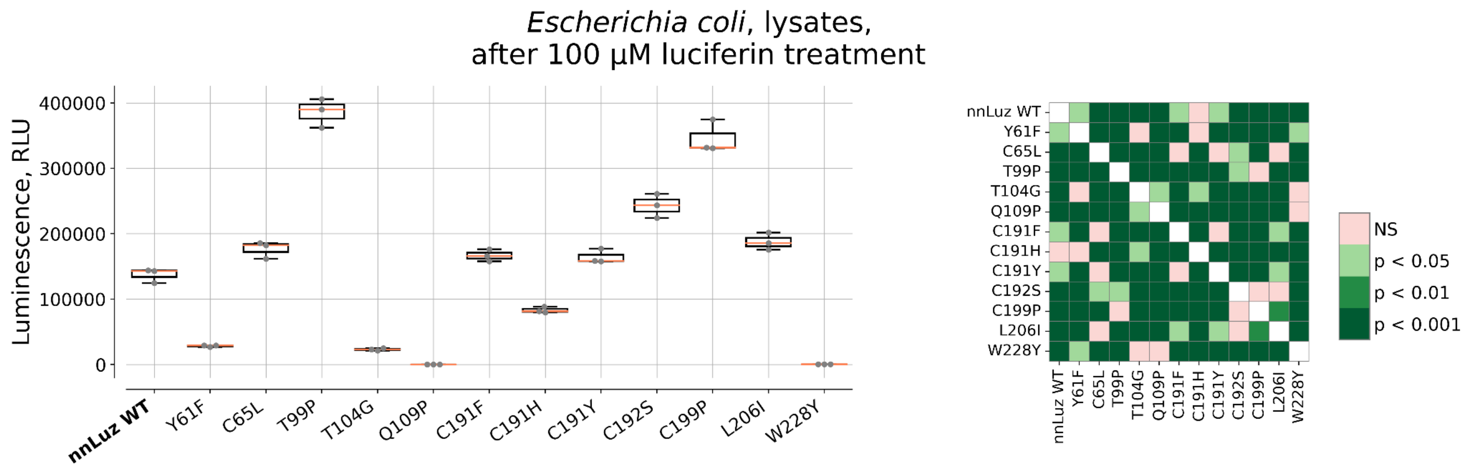

Supplementary Figure 1. Activity of nnLuz mutants obtained in consensus mutagenesis in *E.coli* lysates, after treatment with 100 μM of luciferin. Imaging was performed at room temperature. Maximal brightness of luminescence is shown. Box and whisker plots (left) are accompanied by colour-coded p-values of post-hoc two-sided Conover's test (right) corrected by the step-down method using Sidak adjustments. NS — non-significant. The boxes are the first and the third quartiles, whiskers are the rest of the distribution except outliers, the orange line is the median. N = 3 biologically independent samples per box plot. Kruskal-Wallis H Test: H-statistic = 37.20, p = 2.1e-04.

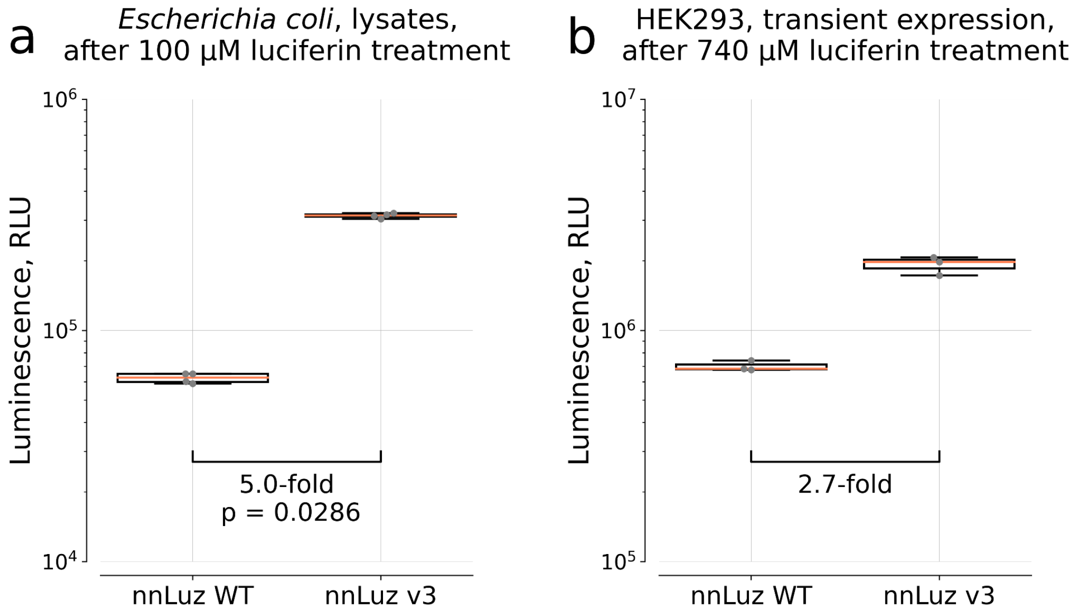

Supplementary Figure 2. Activity of nnLuz v3 (T99P, T192S, A199P) mutant in *E. coli* lysates (**a**) and in transient expression in HEK293 cells (**b**) after treatment with 100 or 740 µM luciferin, respectively. Experiments in *E.coli* lysates were performed at room temperature, in mammalian cells – at 37°C. Maximal brightness of luminescence is shown. The boxes are the first and the third quartiles, whiskers are the rest of the distribution except outliers, the orange line is the median. The difference between mean values and p-values of post-hoc two-sided Mann-Whitney U-tests (if applicable) are indicated below the brackets between the box plots. N = 4 (**a**) or 3 (**b**) biologically independent samples per box plot.

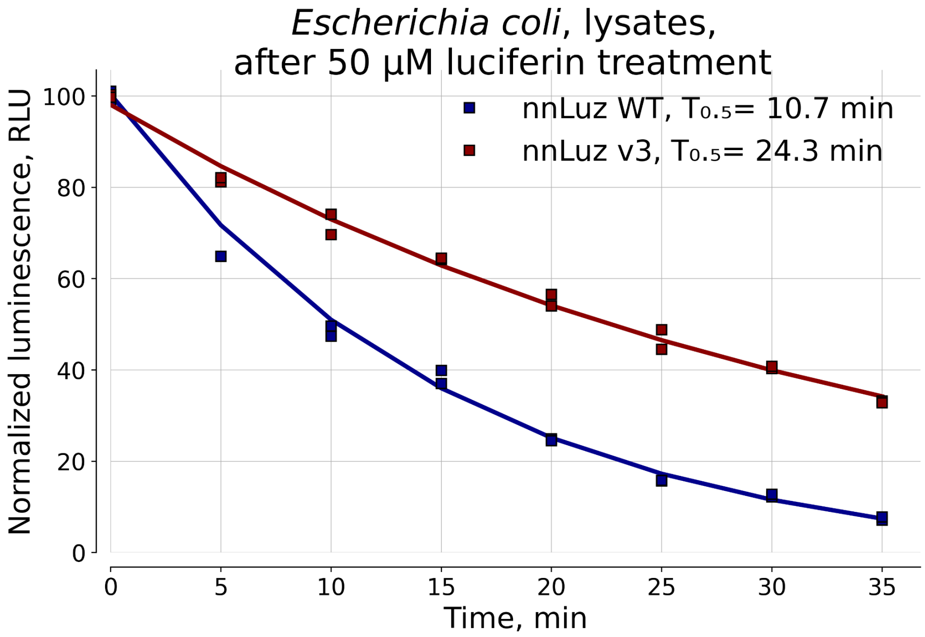

Supplementary Figure 3. Activity of nnLuz WT and nnLuz v3 (T99P, T192S, A199P) in *E. coli* cleared lysates after incubation at 22°C during different periods of time followed by treatment with luciferin (50 µM). Data points are normalised to the data at the time = 0 min. Data is shown as data points (squares) and fitting curves (lines). Data is fitted with an exponential decay equation. N = 2 biologically independent samples.

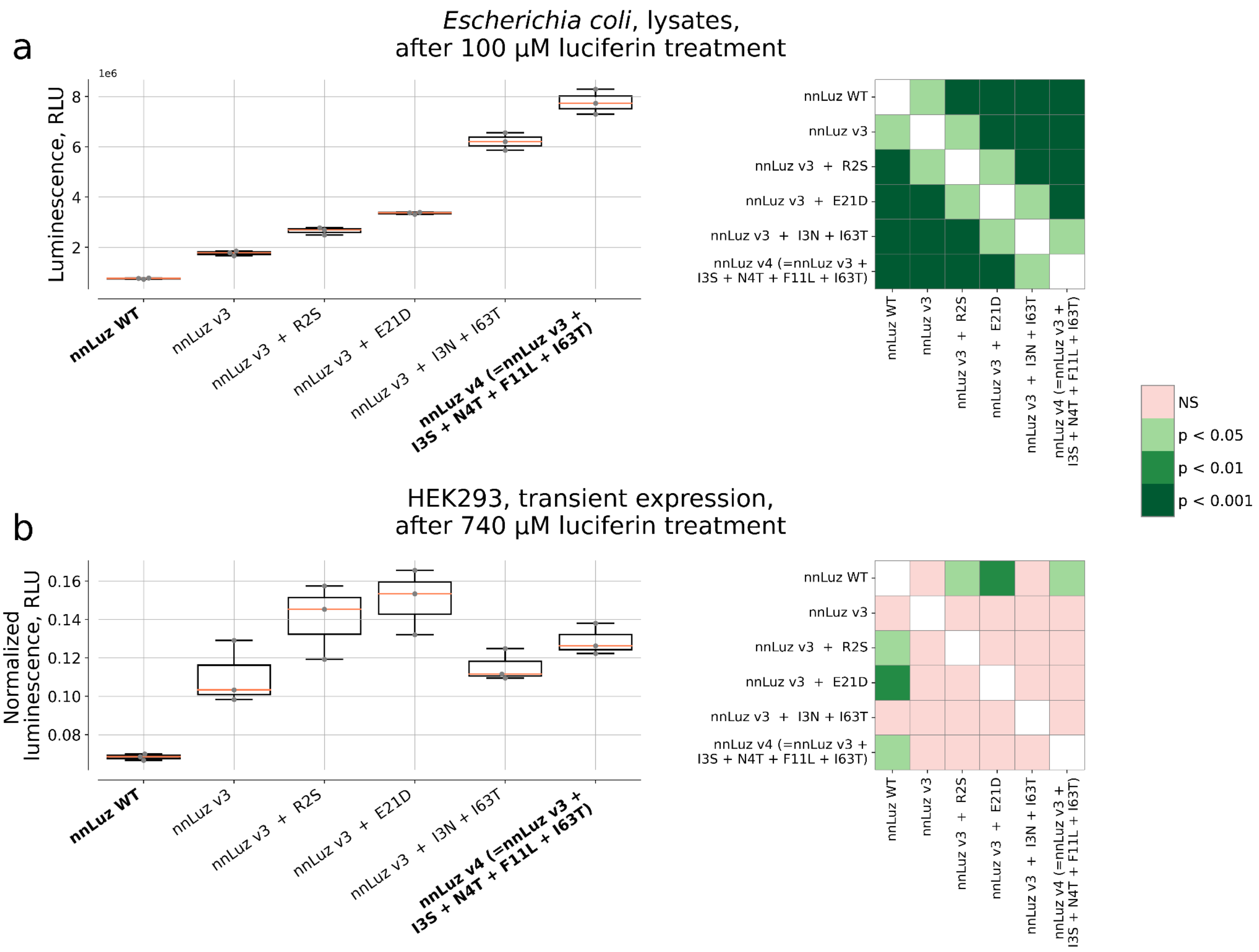

Supplementary Figure 4. Activity of mutants obtained after random mutagenesis of nnLuz v3 in *E. coli* lysates (**a**) and in transient expression in HEK293 cells (**b**) after treatment with luciferin. Maximal brightness of luminescence is shown. In HEK293 luminescence signal was normalised to protein abundance, as measured by HiBiT assay. Box and whisker plots (left) are accompanied by colour-coded p-values of post-hoc two-sided Conover's test (right) corrected by the step-down method using Sidak adjustments. NS — non-significant. The boxes are the first and the third quartiles, whiskers are the rest of the distribution except outliers, the orange line is the median. N = 3 biologically independent samples per box plot. Kruskal-Wallis H Test: H-statistic = 16.58, p = 5.4e-03 (**a**) and H-statistic = 12.84, p = 2.5e-02 (**b**).

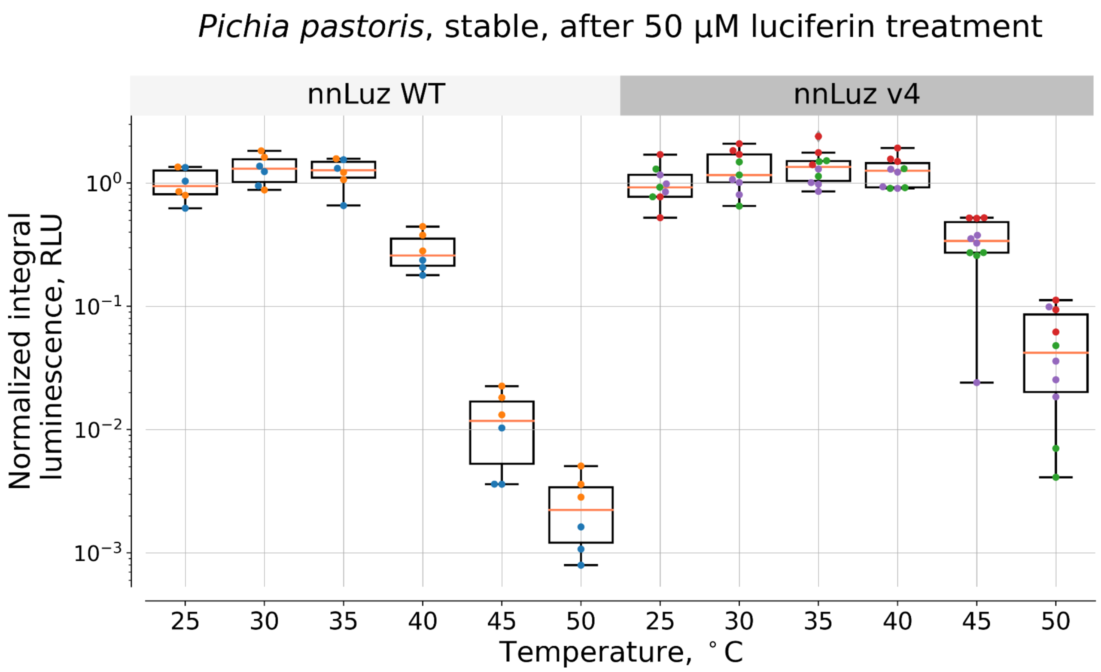

Supplementary Figure 5. Luminescence of yeast cells expressing luciferase variants, after 10 min incubation at different temperatures followed by treatment with luciferin (50 µM). The integral luminescence for 10 min normalised to the integral signal obtained after incubation at 25°C is shown. The boxes are the first and the third quartiles, whiskers are the rest of the distribution except outliers, the orange line is the median. The colour of data points indicates different yeast strains (2 strains for nnLuz WT and 3 strains for nnLuz v4). N = 6-10 biologically independent samples per box plot.

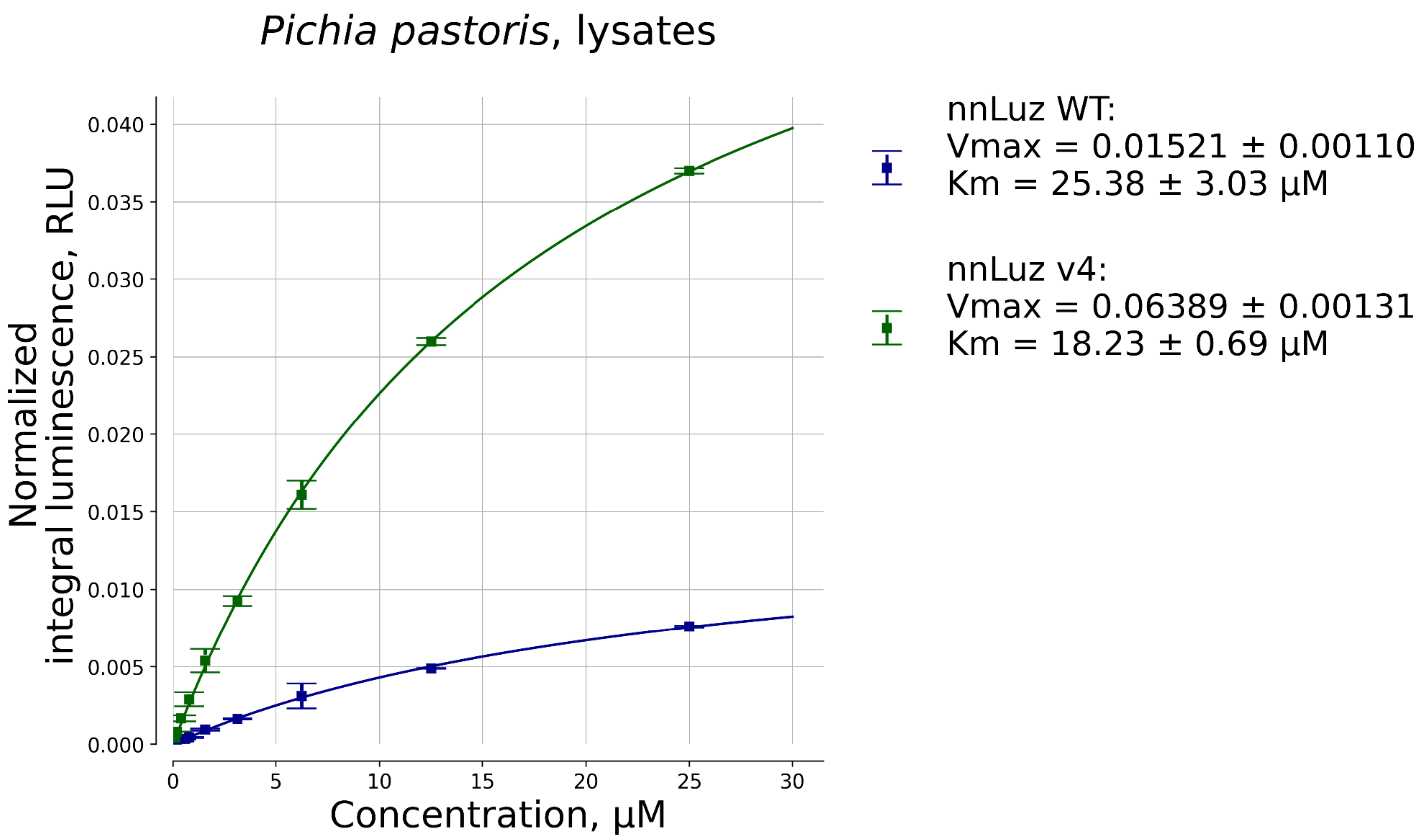

Supplementary Figure 6. Michaelis–Menten curve of nnLuz WT/v4–HiBIT fusions obtained from yeast lysates in the presence of 0.1 - 25 μM fungal luciferin. Data normalized to expression level assessed by HiBit luminescence. Data is shown as data points (squares) ± SD (whiskers) and fitting curves (lines). Data is fitted with a Michaelis-Menten equation. N = 3 biologically independent samples per box plot.

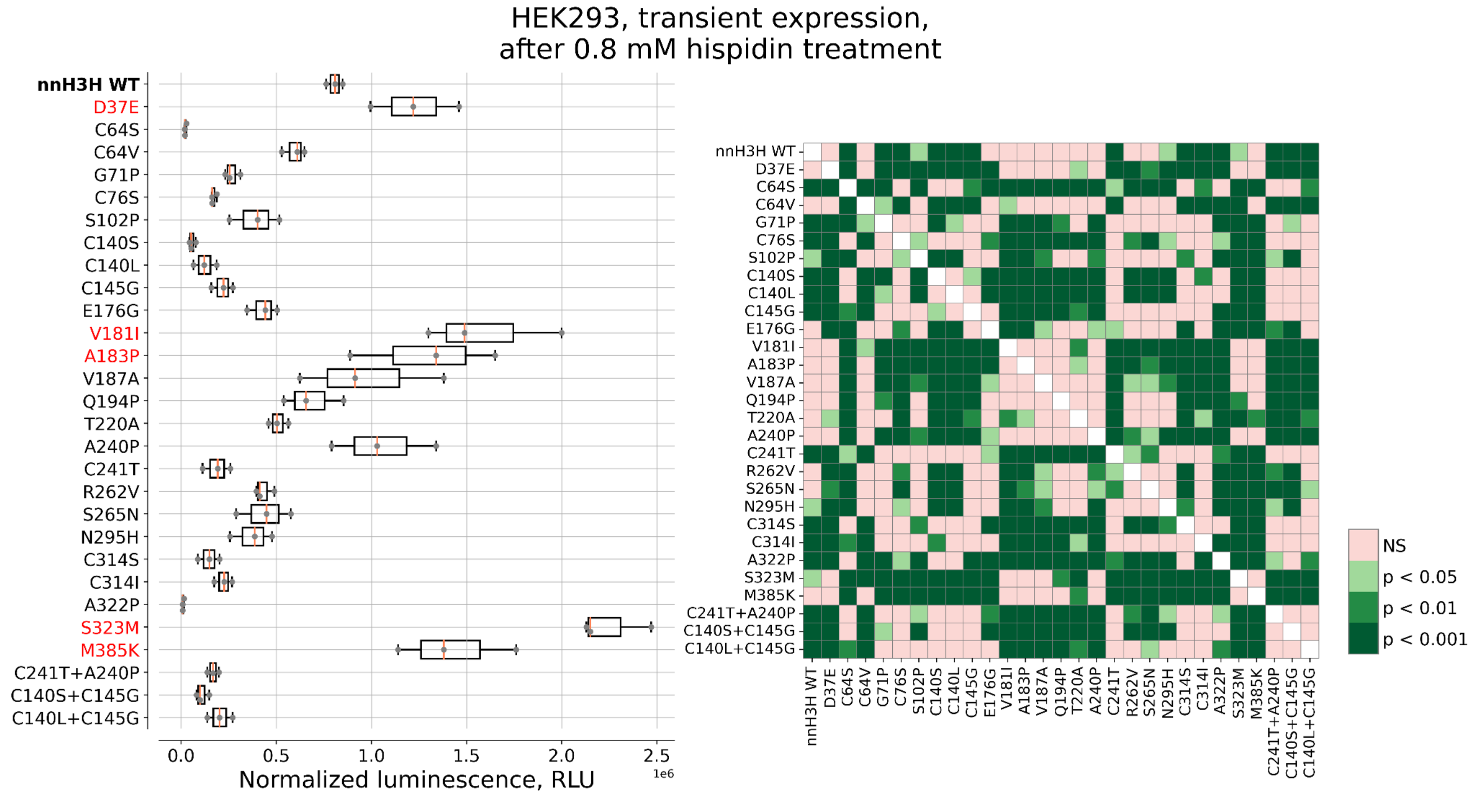

Supplementary Figure 7. Activity of mutants obtained in consensus mutagenesis of nnH3H. Transient co-expression of depicted nnH3H mutants with nnLuz v3 in HEK293 cells, luminescence measured after addition of 0.8 mM hispidin. Luminescence signal was normalised to protein abundance, as measured by HiBiT assay. Red labels correspond to mutations with improved luminescence. Box and whisker plots (left) are accompanied by colour-coded p-values of post-hoc two-sided Conover's test (right) corrected by the step-down method using Sidak adjustments. NS — non-significant. The boxes are the first and the third quartiles, whiskers are the rest of the distribution except outliers, the orange line is the median. N = 3 biologically independent samples per box plot. Kruskal-Wallis H Test: H-statistic = 82.22, p = 3.1e-07.

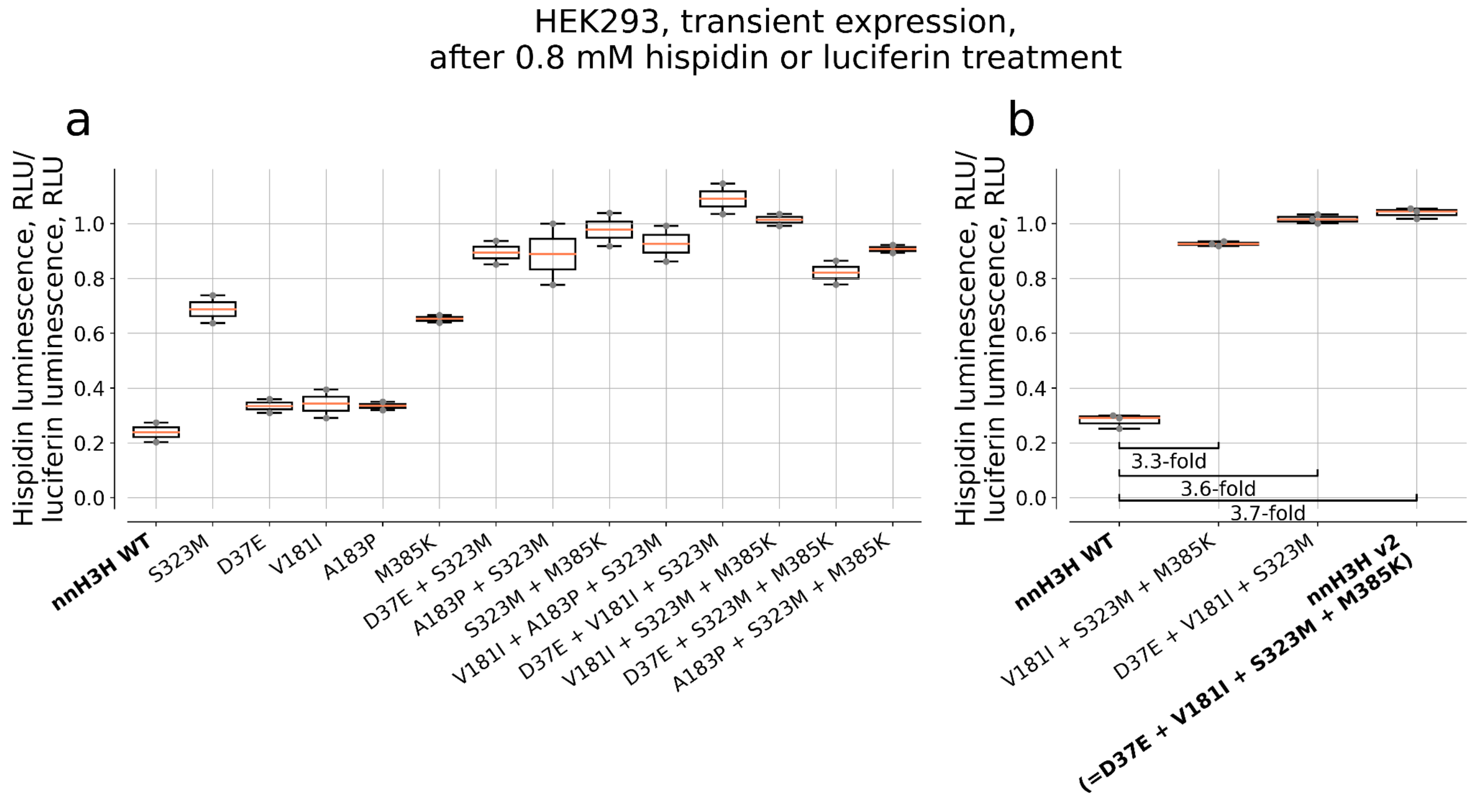

Supplementary Figure 8. Activity of nnH3H mutants in transient expression with nnLuz v3 in HEK293 after treatment with 0.8 mM of luciferin or hispidin. **a.** Combinatorial mutation analysis of nnH3H. N = 2 biologically independent samples per box plot. **b.** Follow-up experiment to assess selected combinations of mutations. N = 3 biologically independent samples per box plot. The boxes are the first and the third quartiles, whiskers are the rest of the distribution except outliers, the orange line is the median.

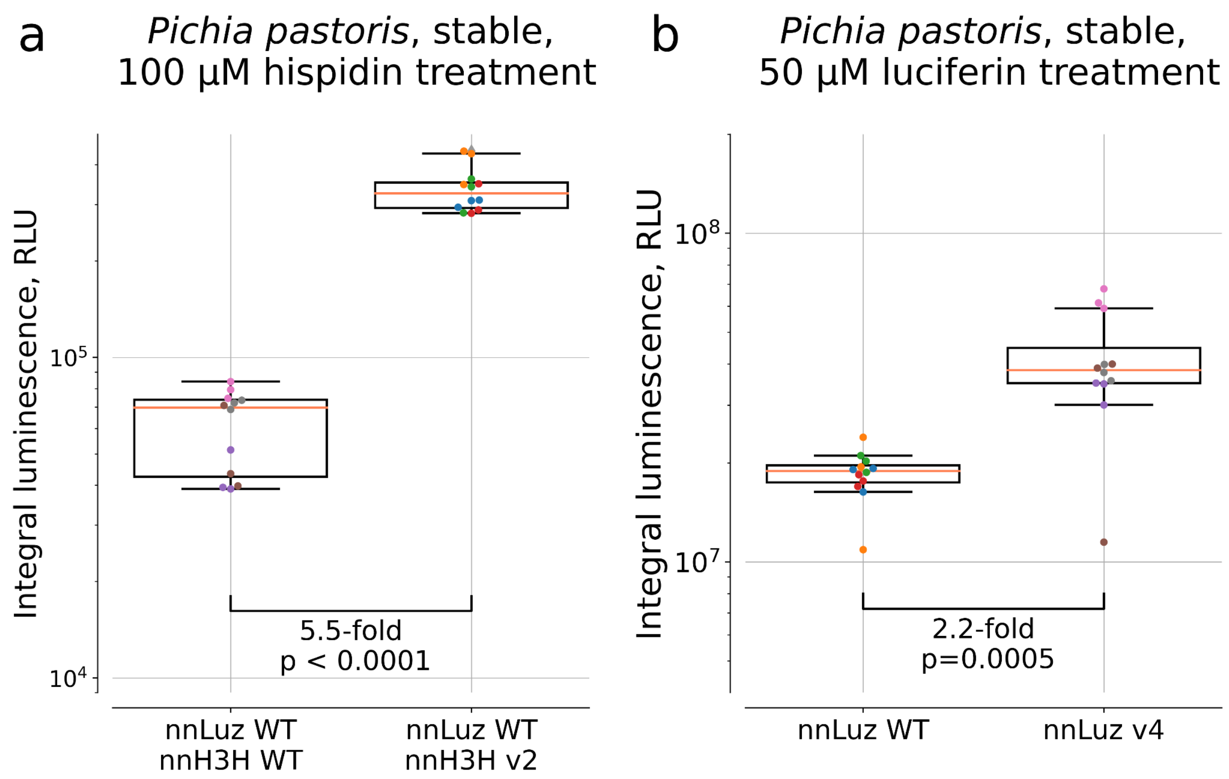

Supplementary Figure 9. Comparison of WT and improved enzymes of nnH3H and nnLuz in *P. pastoris*. Luminescence was assayed after adding 100 µM hispidin (**a**) and 50 µM luciferin (**b**). Integral signal was collected for 20 (**a**) and 10 min (**b**). The boxes are the first and the third quartiles, whiskers are the rest of the distribution except outliers, the orange line is the median. The colour of data points indicates different yeast strains (4 per box plot). N = 12 biologically independent samples per box plot. The difference between mean values and p-values of post-hoc two-sided Mann-Whitney U-tests are indicated below the brackets between the box plots, p = 3.7e-05 (**a**), p = 4.8e-04 (**b**).

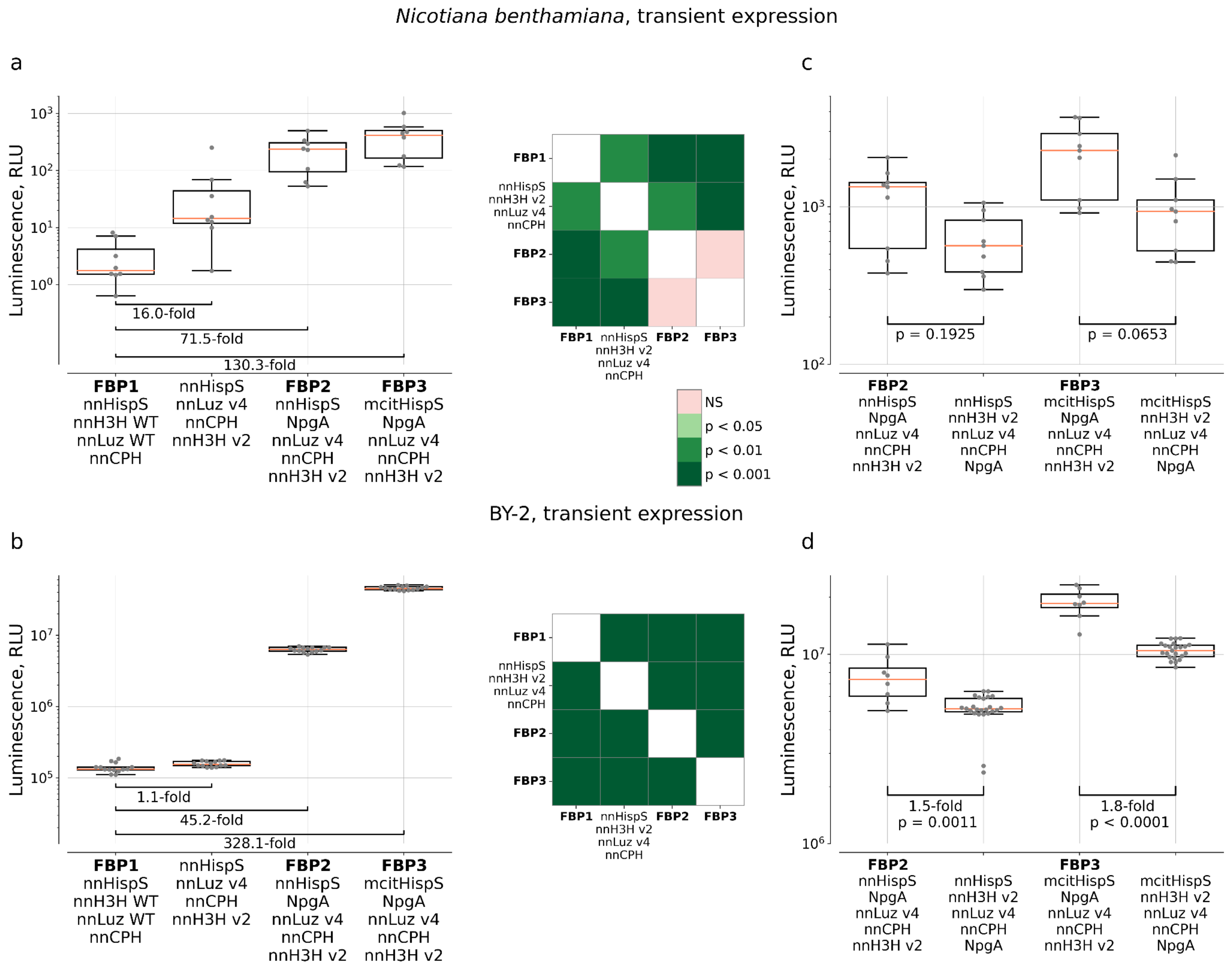

Supplementary Figure 10. Comparison of FBP versions, and assembly variants with different order of transcription units. The experiments were performed in *N.benthamiana* leaves **(a, c)** and BY-2 plant cell packs **(b, d)**. Named versions refer to plasmids shown on Extended Data Figure 4 **(a, b)**. The order of gene names in the labels of the X axis corresponds to the order of transcription units in multi-gene plasmids. Box and whisker plots are accompanied by colour-coded p-values of two-sided Conover's test corrected by the step-down method using Sidak adjustments (**a**, **b**). NS — non-significant. The boxes are the first and the third quartiles, whiskers are the rest of the distribution except outliers, the orange line is the median. N = 8 (**a**) or 9 (**c**) leaves per box plot , N = 14 (**b**) or 8-24 (**d**) cell packs per box plot . The difference between mean values is indicated below the brackets between the box plots, in (**c**, **d**) supplied with p-value of post-hoc two-sided Mann-Whitney U-test. In (**d**) p = 1.1e-03 and 2.5e-07 for FBP2 vs nnHispS + nnH3H v2 + nnLuz v4 + nnCPH + NpgA and FBP3 vs mcitHispS + nnH3H v2 + nnLux v4 + nnCPH + NpgA, respectively. Kruskal-Wallis H Test: H-statistic = 23.59, p = 3.0e-5 (**a**) and H-statistic = 48.10, p = 2.0e-10 (**b**).

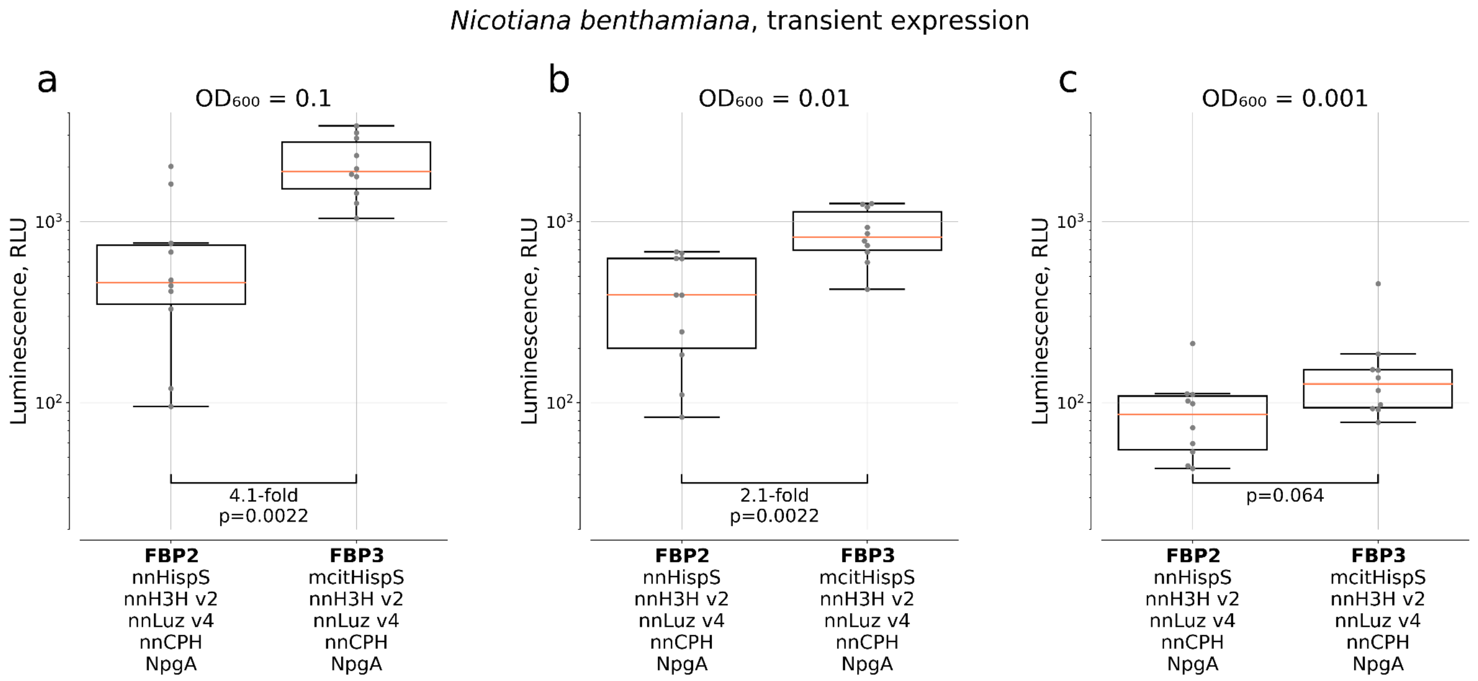

Supplementary Figure 11. Comparison of FBP2 and FBP3 pathways in *N. benthamiana* leaves infiltrated with agrobacterial suspensions of different optical densities. OD_{600} = 0.1 (**a**), 0.01 (**b**) or 0.001 (**c**). The boxes are the first and the third quartiles, whiskers are the rest of the distribution except outliers, the orange line is the median. N = 10 leaves per box plot. The difference between mean values and p-values of post-hoc two-sided Mann-Whitney U-tests are indicated below the brackets between the box plots.

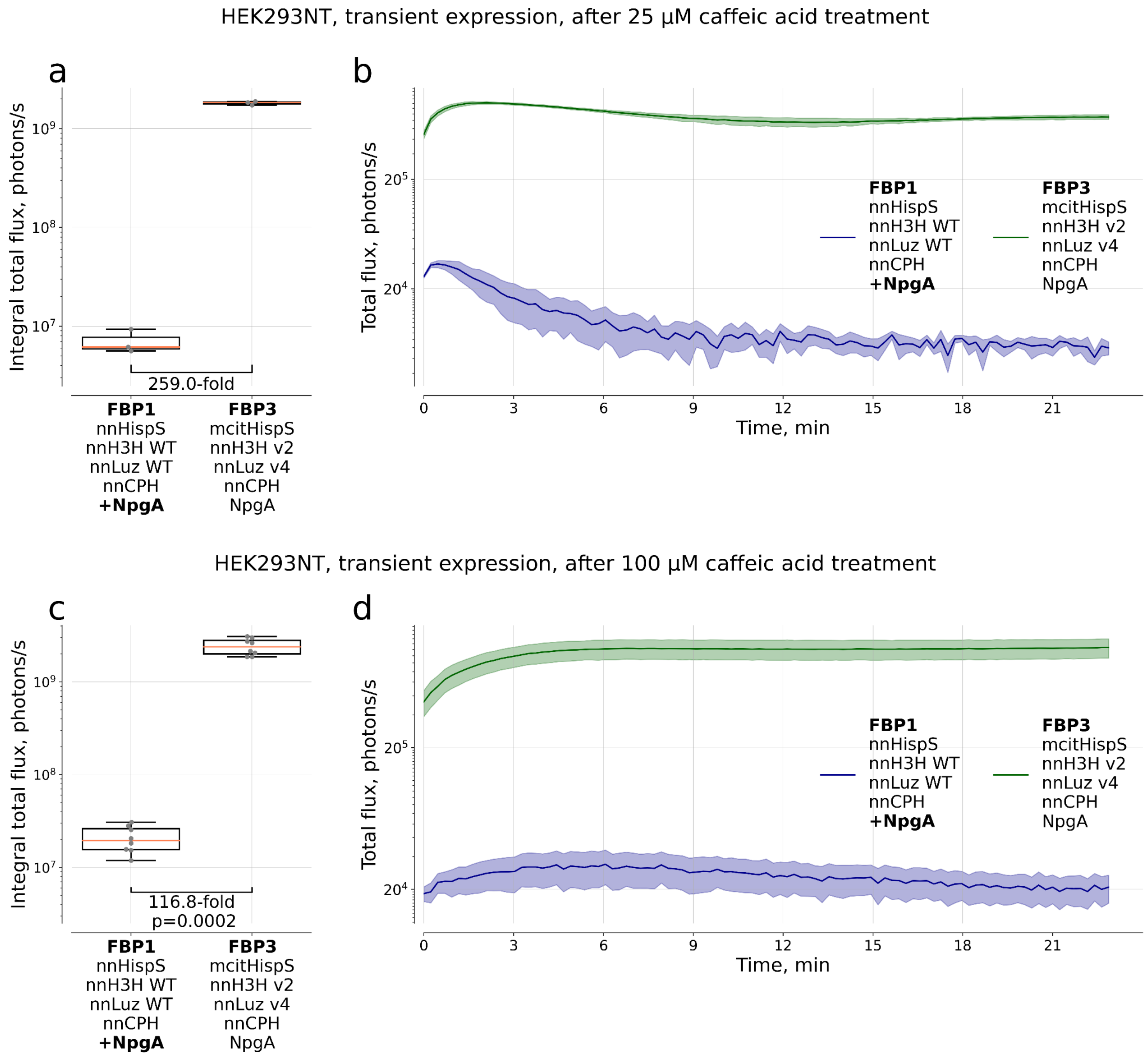

Supplementary Figure 12. FBP1 and FBP3 pathways comparison in transient expression assay in HEK293NT cells after 25 µM (a,b) and 100 µM (c, d) caffeic acid treatment. (**a, c**) Integral signal for 23 minutes. The boxes are the first and the third quartiles, whiskers are the rest of the distribution except outliers, the orange line is the median. N = 3 (**a**) and 8 (**c**) biologically independent replicates per box plot. The difference between mean values and p-values of post-hoc two-sided Mann-Whitney U-tests (if applicable) are indicated below the brackets between the box plots. (**b,d**) Kinetics. Data shown as mean (solid line) ± SD (area around the solid line).

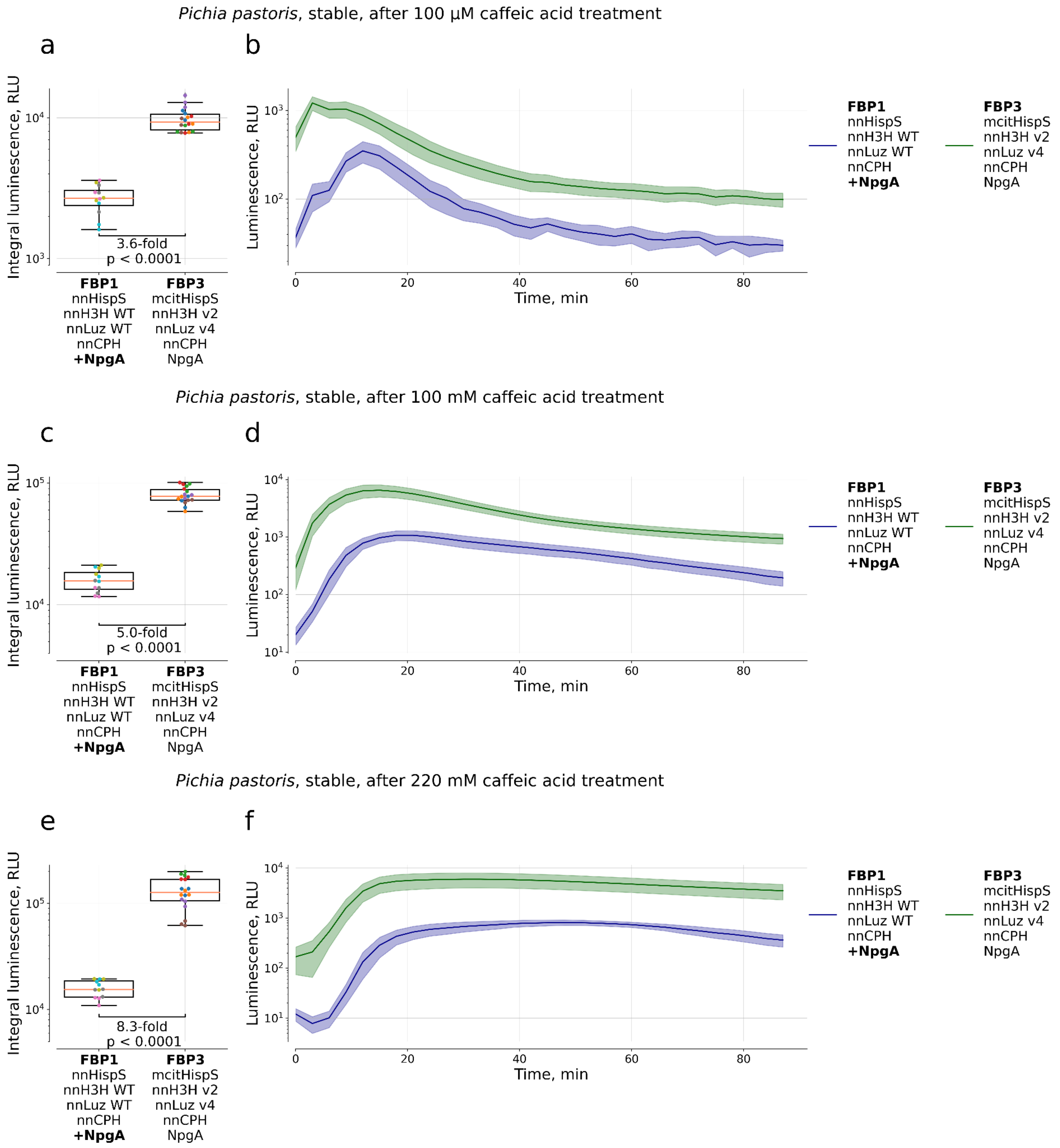

Supplementary Figure 13. The comparison of *P. pastoris* strains expressing FBP1 or FBP3 pathways after 100 µM (a, b), 100 mM (c, d) and 220 mM (e, f) caffeic acid treatment. (**a**, **c**, **e**) Integral signal for 90 minutes. The boxes are the first and the third quartiles, whiskers are the rest of the distribution except outliers, the orange line is the median. The colour of data points indicates different yeast strains (4 yeast strains for FBP1 and 6 for FBP3). The difference between mean values and p-values of post-hoc two-sided Mann-Whitney U-tests are indicated below the brackets between the box plots, p = 5.0e-06 (**a**), p = 5.0e-06 (**c**), p = 5.0e-06 (**e**). N = 12 or 18 biologically independent replicates for FPB1 or FBP3 in each panel, respectively. (**b**,**d**, **f**) Kinetics. Data shown as mean (solid line) ± SD (area around the solid line).

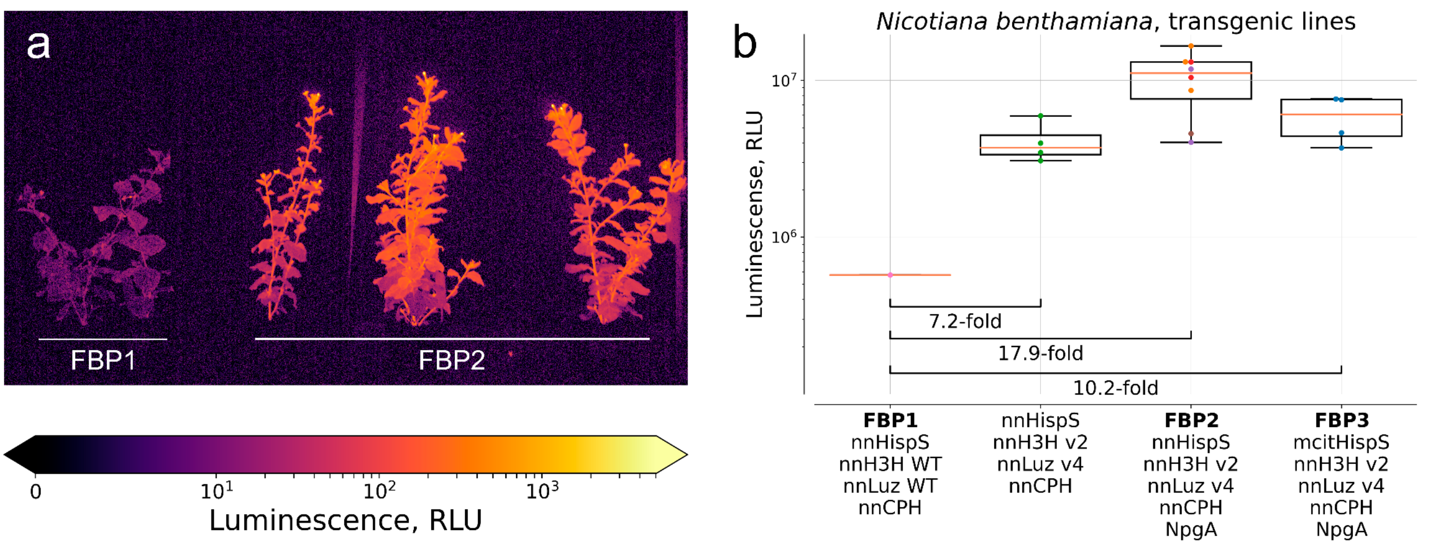

Supplementary Figure 14. Imaging of 9-week-old transgenic *N. benthamiana*. The FBP1 is the plant line NB021 reported in ref [1]. The photo, captured with ISO 400 and exposure of 30 sec (**a**) and integral luminescent signal from whole plants (**b**). It is interesting to note that when FBP1 is not co-expressed with NpgA, *N. benthamiana* plants generally produce significantly more light than *N. tabacum* plants. We interpret this as evidence of higher levels of endogenous PPTase activity in *N. benthamiana*.

The boxes are the first and the third quartiles, whiskers are the rest of the distribution except outliers, the orange line is the median. The colour of data points indicates different plant lines: 1 line for FBP1 (N = 1 plant), 1 line for nnHispS + nnH3H v2 + nnLuz v4 + nnCPH (N = 4 plants), 4 lines for FBP2 (N = 8 plants), 1 line for FBP3 (N = 4 plants).The difference between mean values is indicated below the brackets between the box plots.

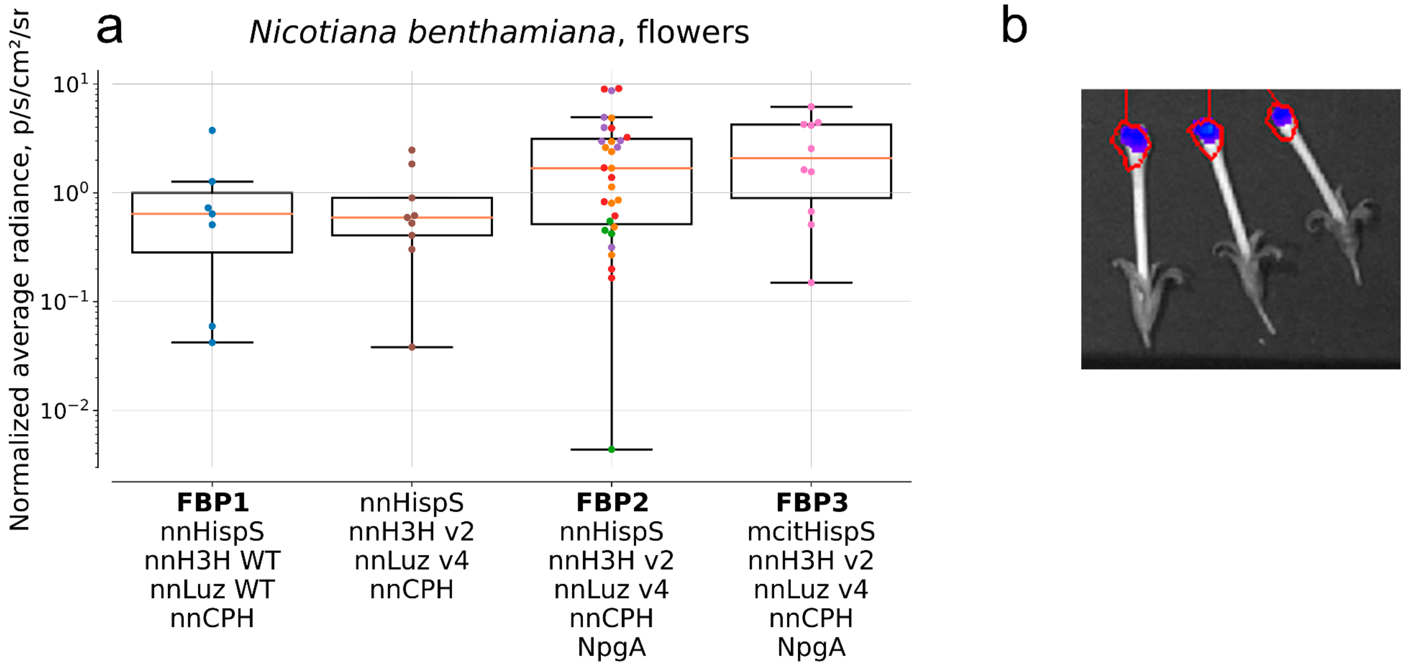

Supplementary Figure 15. Imaging of flowers of 7-week-old transgenic *N. benthamiana*. The FBP1 is the plant line NB021 reported in ref [1]. The boxes are the first and the third quartiles, whiskers are the rest of the distribution except outliers, the orange line is the median. The colour of data points indicates different plant lines: NB021 line for FBP1 (N = 7 flowers), 1 line for nnHispS + nnH3H v2 + nnLuz v4 + nnCPH (N = 9 flowers), 4 lines for FBP2 (N = 31 flowers), 1 line for FBP3 (N = 10 flowers). Kruskal-Wallis H Test: H-statistic = 6.48, p = 0.09. (**c**) Example of selected ROI of corona of flowers.

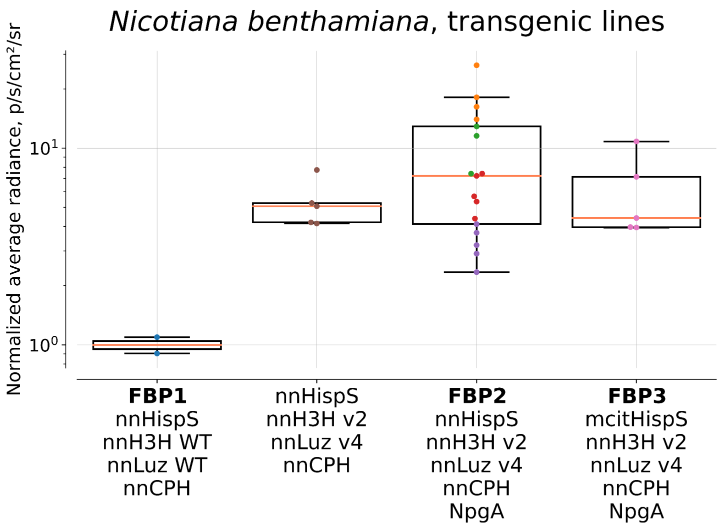

Supplementary Figure 16. Imaging of leaves taken from the top of 7-week-old transgenic *N. benthamiana*.The FBP1 is the plant line NB021 reported in ref [1]. The boxes are the first and the third quartiles, whiskers are the rest of the distribution except outliers, the orange line is the median. The colour of data points indicates different plant lines: NB021 line for FBP1 (N = 2 leaves), 1 line for nnHispS + nnH3H v2 + nnLuz v4 + nnCPH (N = 5 leaves), 4 lines for FBP2 (N = 17 leaves), 1 line for FBP3 (N = 5 leaves). Kruskal-Wallis H Test: H-statistic = 5.61, p = 0.13.

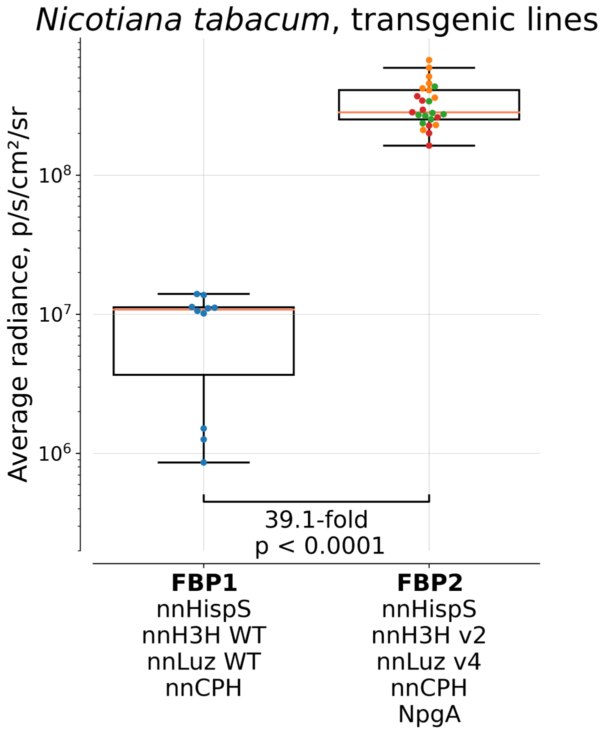

Supplementary Figure 17. Average luminescence of leaves taken from the top of 7.5-week-old *N. tabacum* expressing FBP1 or FBP2 pathways. The FBP1 is the plant line NT001 reported in ref [1]. The boxes are the first and the third quartiles, whiskers are the rest of the distribution except outliers, the orange line is the median. The colour of data points indicates different plant lines (FBP1 - NT001, for FBP2 - 3 lines per box plot); N = 10 or 25 leaves for FBP1 or FBP2, respectively. The difference between mean values and p-values of post-hoc two-sided Mann-Whitney U-test are indicated below the brackets between the box plots, p = 5.0e-06.

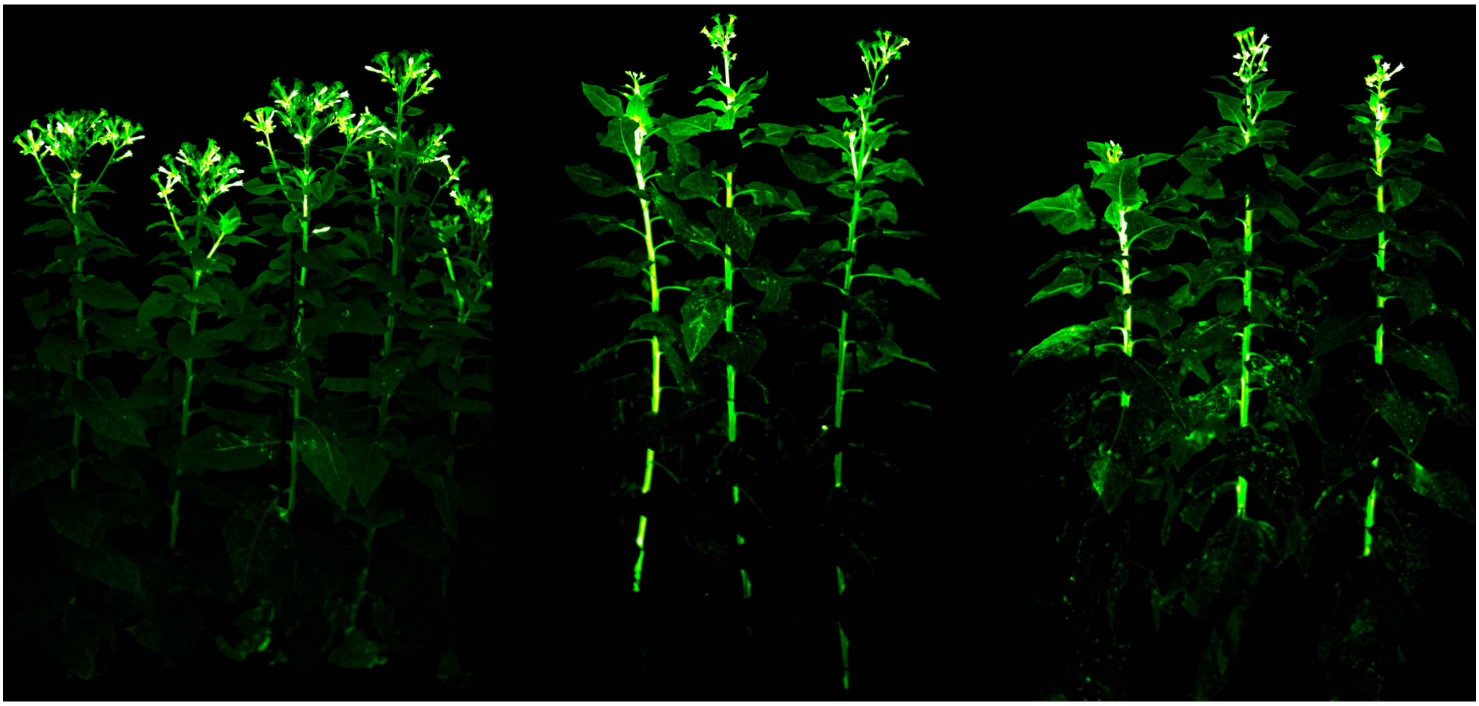

Supplementary Figure 18. Imaging of 9-week-old *N. tabacum* plants representing 3 different lines expressing FBP2 pathway. ISO 400, exposure 30 sec (see Methods).

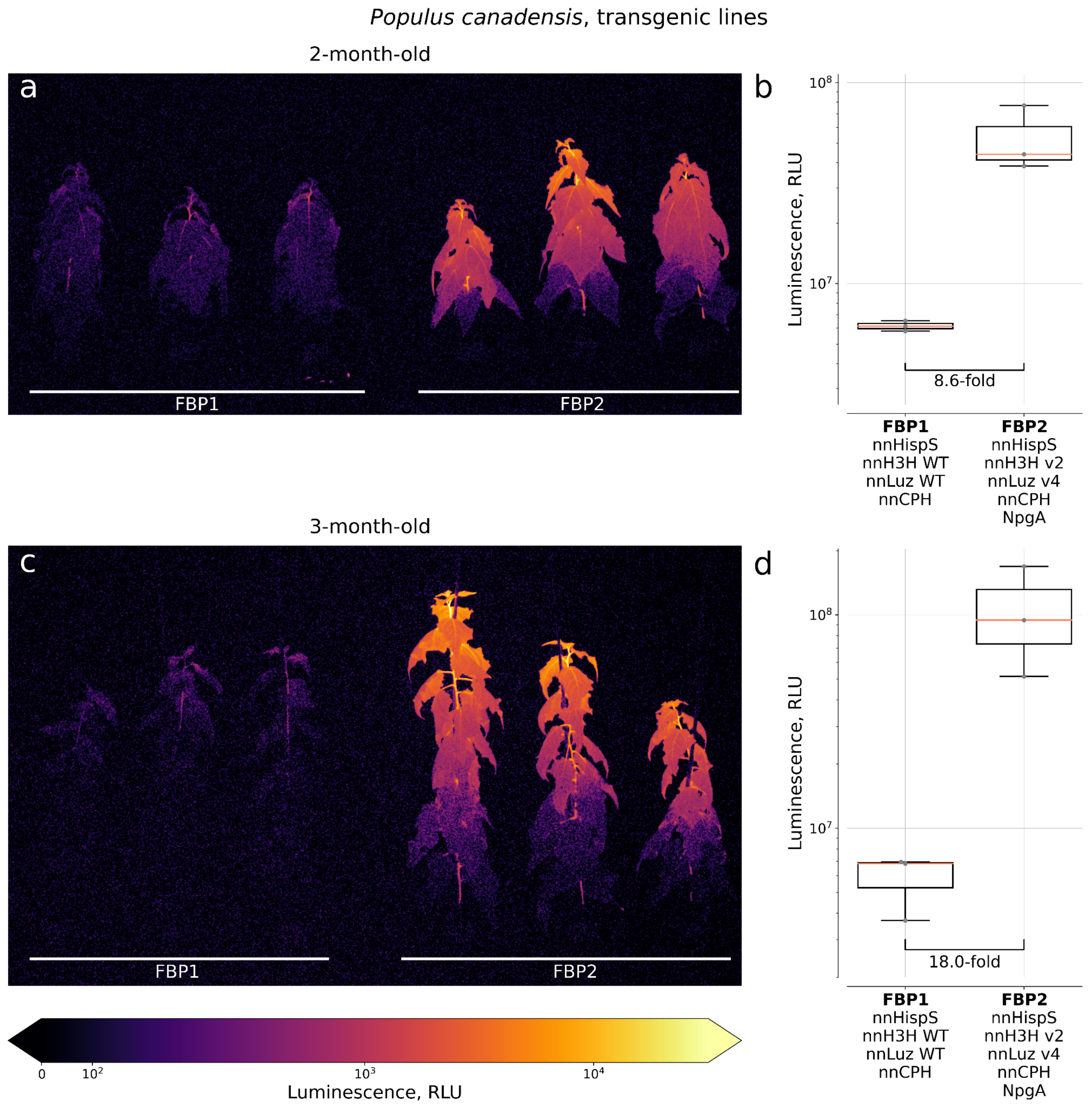

Supplementary Figure 19. Imaging of *P. canadensis* transformed with FBP1 or FBP2 pathways. The photo of 2-month-old (**a**) or 3-month-old plants (**c**) and corresponding integral luminescence signal from the whole plants (**b**, **d**). Photos were captured with ISO 20000 and exposure of 30 sec. The boxes are the first and the third quartiles, whiskers are the rest of the distribution except outliers, the orange line is the median. N = 3 plants per box plot. The difference between mean values is indicated below the brackets between the box plots.

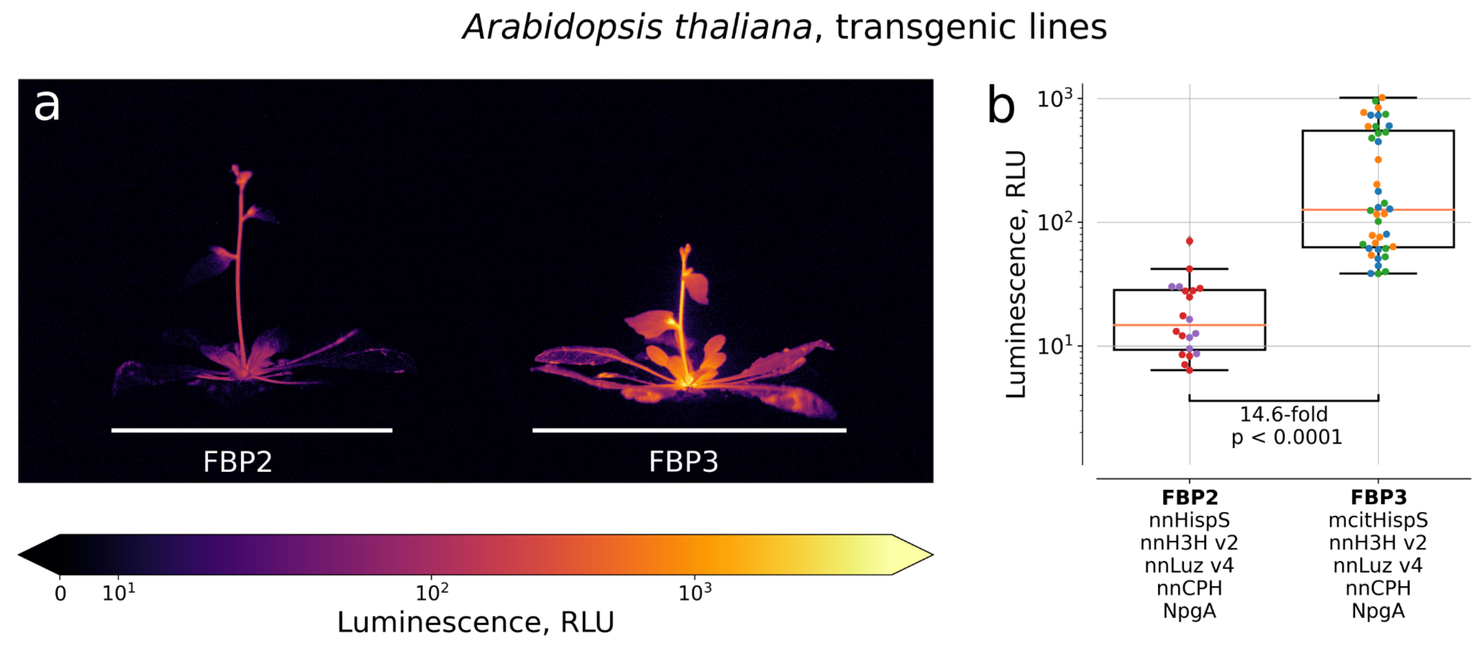

Supplementary Figure 20. Average luminescence of leaves of 6-week-old *A.thaliana* transformed with FBP2 and FBP3 pathways. The photo (ISO 400, exp - 30 sec)(**a**) and average luminescence of leaves(**b**). The boxes are the first and the third quartiles, whiskers are the rest of the distribution except outliers, the orange line is the median. The colour of data points indicates different plant lines (2 lines for FBP2, 3 lines for FBP3 per box plot); N = 20 or 40 leaves for FBP2 or FBP3, respectively. The difference between mean values and p-value of post-hoc two-sided Mann-Whitney U-test are indicated below the brackets between the box plots, p = 1.8e-09.

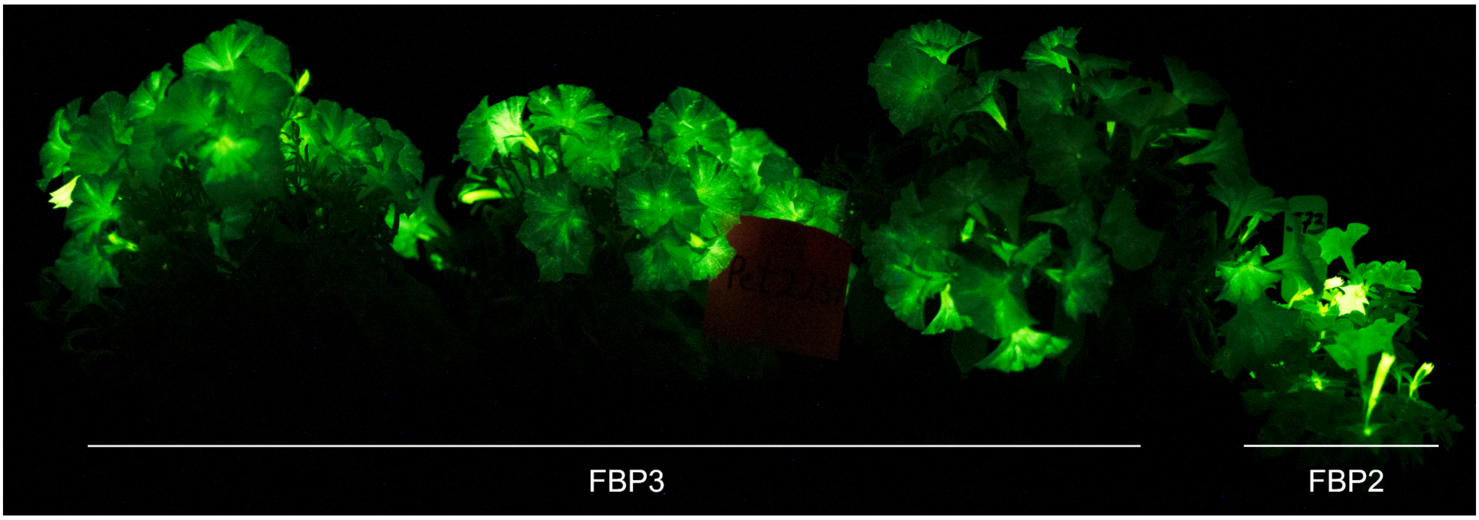

Supplementary Figure 21. Imaging of *P. hybrida* transformed with FBP2 and FBP3 pathways.
ISO 3200, exposure – 30 sec.

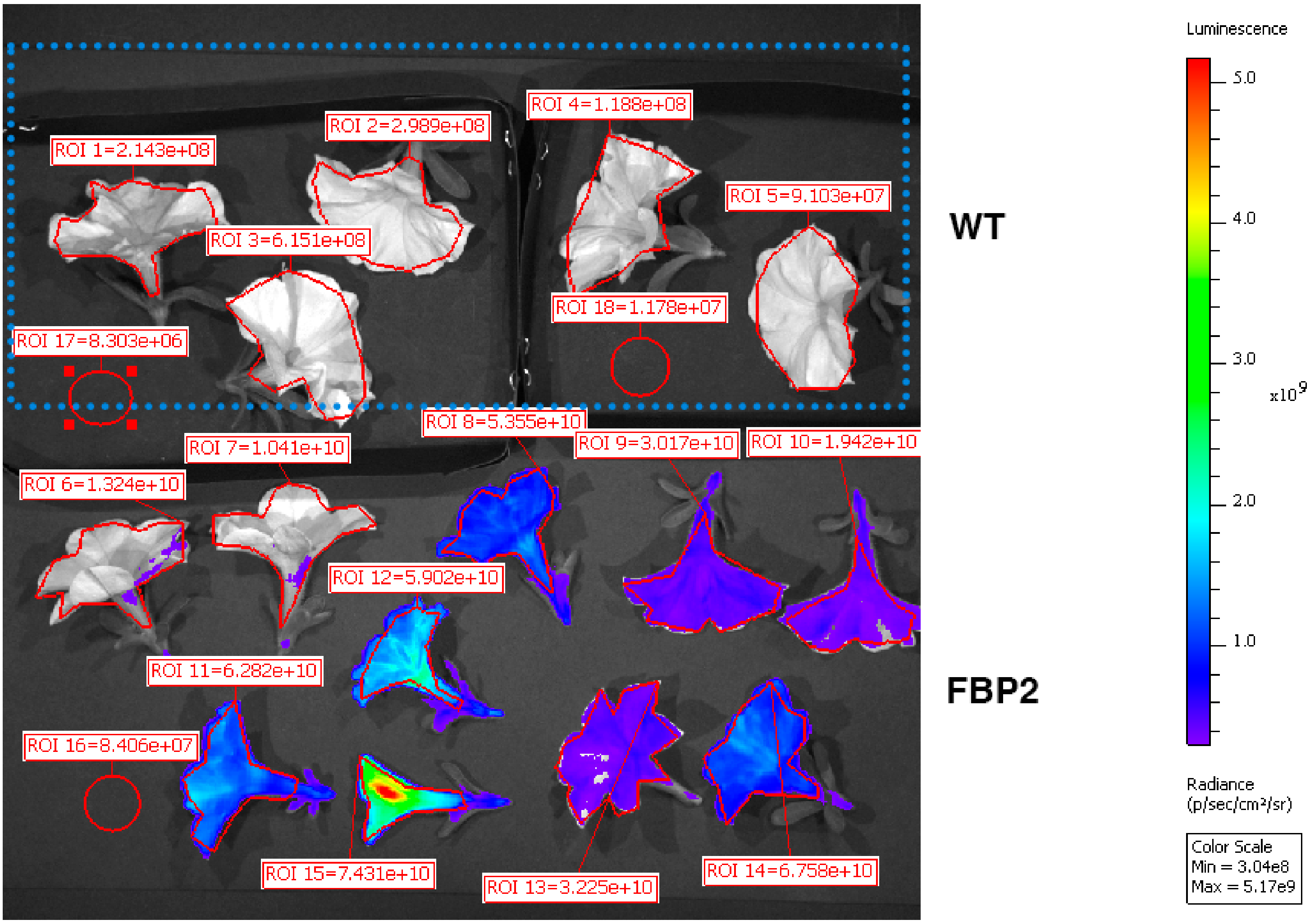

Supplementary Figure 22. Imaging of flowers of *P. hybrida* transformed with FBP2 pathway (out of the black paper box), in the black paper box - control flowers of *P. hybrida* WT, values next to ROI represent average radiance (p/s/cm²/sr).

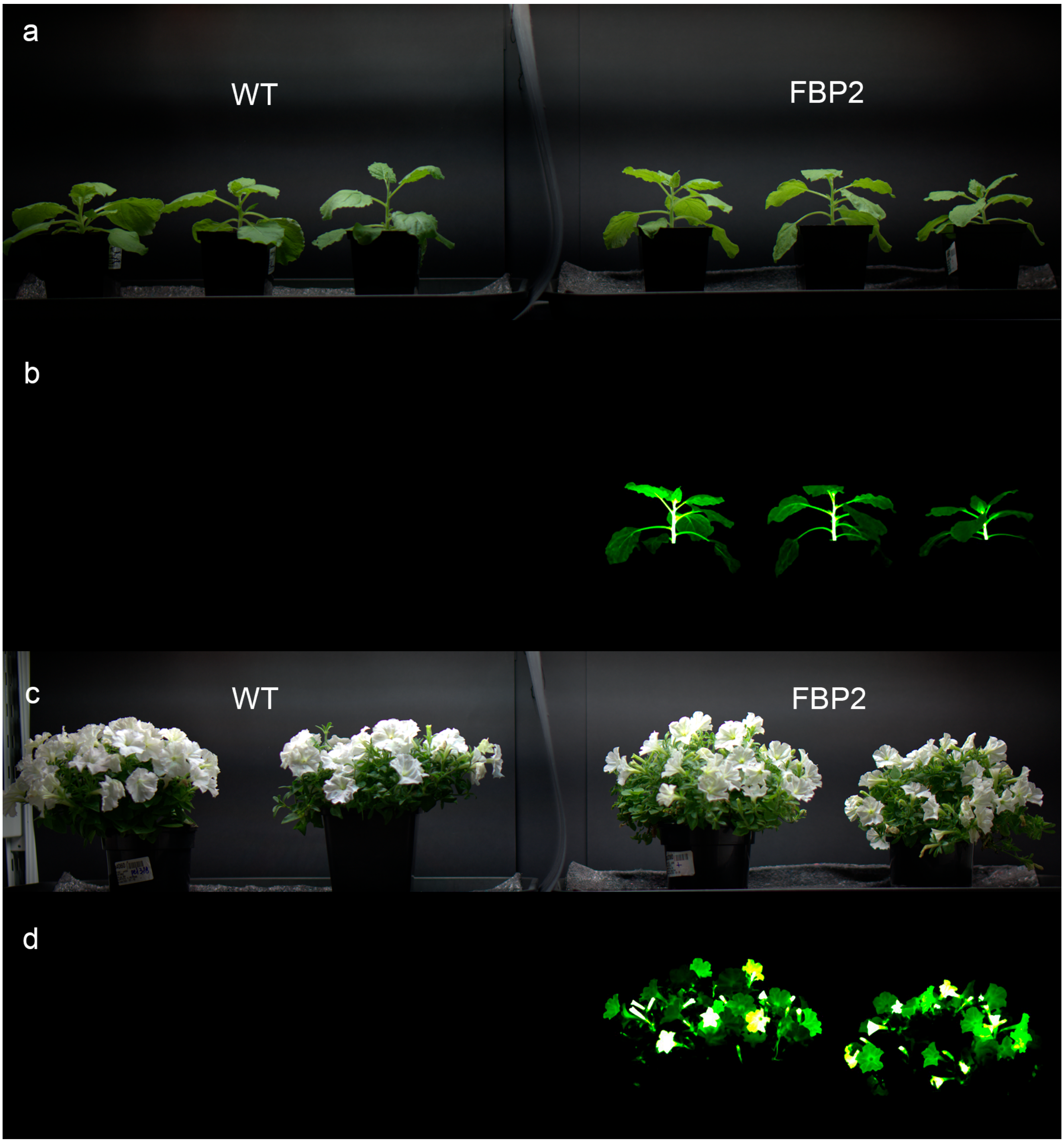

Supplementary Data Figure 23. Phenotype of glowing and wild-type *Nicotiana benthamiana* and *Petunia hybrida* plants. Potted wild-type and glowing plants of the same age in ambient light (a, c) and in the dark (b, d).

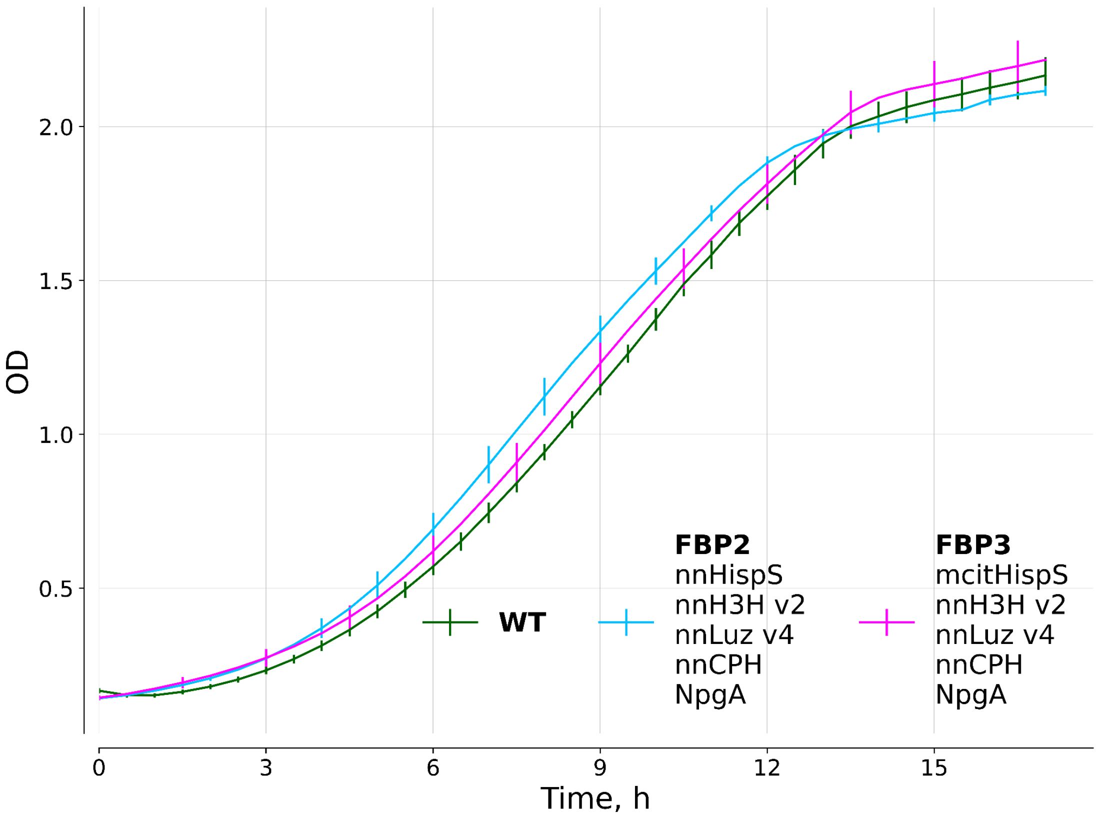

Supplementary Figure 24. The growth of the yeast strain *P. pastoris* GS115 WT and strains expressing FBP1 or FBP3 pathways. N = 1 yeast strain for WT, 1 for FBP2 and 2 yeast strains for FBP3. N = 3, 6 and 12 biologically independent replicates for WT, FBP2 and FBP3, respectively. Data shown as mean (solid line) ± SD (whiskers).

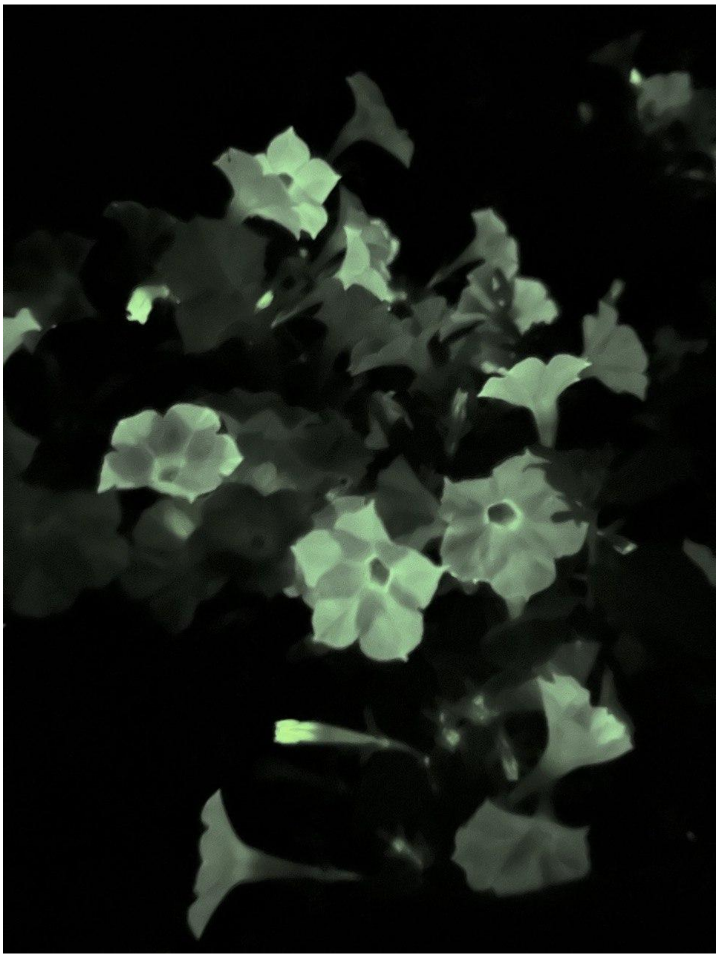

Supplementary Figure 25. A non-edited photo of FBP3-expressing T3 petunia line, shot on iPhone 14 Pro.

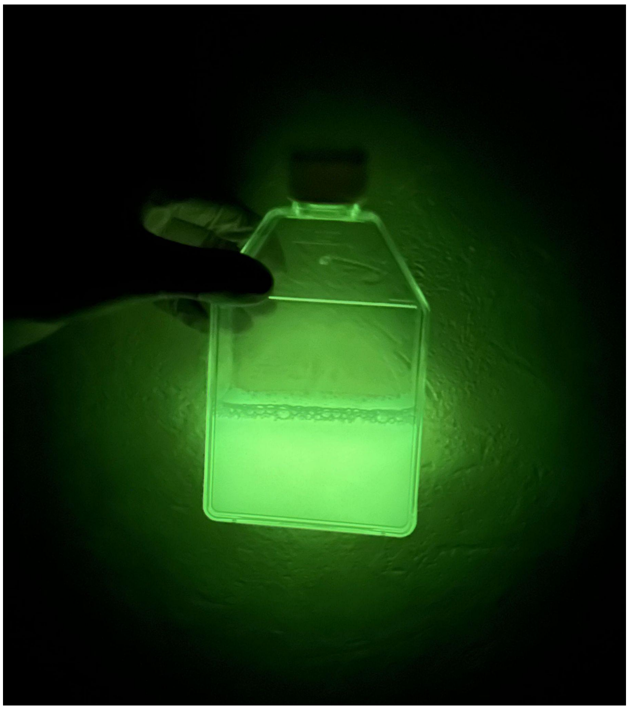

Supplementary Figure 26. A non-edited photo of FBP3-expressing yeast strain in media supplemented with caffeic-acid, shot on iPhone 14.

Supplementary Video captions

Supplementary Video 1. A non-edited video of FBP3-expressing yeast strain in media supplemented with caffeic-acid, shot on iPhone 14. 26 frames per second, 1920x1080 px.

Supplementary Video 2. Video-rate luminescence imaging of transgenic plants expressing FBP2.

Supplementary Video 3. A non-edited video of FBP3-expressing T3 petunia line, shot on iPhone 13 Pro. 27 frames per second, 1920x1080 px.

Supplementary Table 1. Plasmids used in this study.

**Gene**	**Plants**	**HEK293NT**	***P. pastoris***
NpgA	pX131<br>https://benchling.com/s/seq-j7hk6Mo26QwL2oWED3un?m=slm-HdHkCqJfryAnOv6asct4		
CPH	pX020<br>https://benchling.com/s/seq-6LZLUoXSSOLiGcLsf4Kh?m=slm-EYDYDjEJRJS3k5i8xCim<br><br>Plasmid from the Ref [1]		
nnHispS	bent<br>pX021<br>https://benchling.com/s/seq-dqVnzNzzdb1O8UOlwsyO?m=slm-idFQm0ekAOkLIE1D6Wn8<br><br>pX022<br>https://benchling.com/s/seq-HfeqUXOckkj1jMpb8yjL?m=slm-KGFqWOofY1Qg47FNvgoW<br><br>Plasmids from the Ref [1]	hum<br>https://benchling.com/s/seq-byUf6AubNNzHTRvJWeNE?m=slm-ZGRq9MG6HGKrTHLR5MOP	ppas<br>pNK4498<br>https://benchling.com/s/seq-0qFj0EeLuSEPOTReGrGi?m=slm-zpt87PSoadoe2DyTihdO
afHispS from *Armillaria fuscipes*	ppas/bent/hum<br>pNK6009<br>https://benchling.com/s/seq-HpX0quRopNzuXCbkF5Og?m=slm-orj90OTrXMnI4gONdXJF		
agHispS from *Armillaria gallica*	ppas/bent/hum<br>pNK2655<br>https://benchling.com/s/seq-B3s5evCuyPshIQ6CKV74?m=slm-LfzIK93bmq7cDAJLcgSd		
amHispS from *Armillaria mellea*	ppas/bent/hum<br>pNK2656<br>https://benchling.com/s/seq-LDE3DqjzjVztM8DgiQ0P?m=slm-9TE0Rs2JHhX2nvAUdFrT		
aoHispS from *Armillaria ostoyae*	ppas/bent/hum<br>pNK2652<br>https://benchling.com/s/seq-7nLForLwJbaAC979vsFp?m=slm-UCKyNedQWh16FeQoNYe7		
gnHispS from *Guyanagaster necrorhiza*	ppas/bent/hum<br>pNK2719<br>https://benchling.com/s/seq-StnFUnRATVrtB8pA86S2?m=slm-jy2iuDGpOiMtNCcT25uU		
mchHispS from *Mycena chlorophos*	ppas/bent/hum<br>pNK2653<br>https://benchling.com/s/seq-6CYJYzhlFKzsRPW7Rz9V?m=slm-FJDKAWwsRYmNn1htk0BL		
mcitHispS from *Mycena citricolor*	ppas/bent/hum<br>pNK2657<br>https://benchling.com/s/seq-xd4LOOhTXWIL5RMO7yqa?m=slm-HDLsqpEWOQXnf12rOno5		
ngHispS from *Neonothopanus gardneri*	ppas/bent/hum<br>pNK2842<br>https://benchling.com/s/seq-1M2h5oTlyutLITu1F2ai?m=slm-VA5fzuD7R0z9I6mJjglf		
psHispS #1 from *Panellus stipticus*	ppas/bent/hum<br>pNK2659<br>https://benchling.com/s/seq-VCk6vPdlaky1ZplEvAFl?m=slm-7rKLyhfDI3xwQysqyZ0N		

<table>
<tr><td>psHispS #2 from Panellus stipticus</td><td colspan="3">ppas/bent/hum
pNK2660
https://benchling.com/s/seq-1VmaBYvYwc93bfHoWYN8?m=slm-1N6afQYNHnz3xnA2hD8j</td></tr>
<tr><td>nnH3H WT</td><td>bent
pX019
https://benchling.com/s/seq-ZVjoLOvL9ipQb0Jdx0PW?m=slm-UEsBpjNhXxwZpv9tta4F

Plasmid from the Ref [1]</td><td>hum
pNK180
https://benchling.com/s/seq-ZGuO24Ks9ONH5OuPDrmR?m=slm-h01x8hemK2Co5EiA5O1V</td><td>ppas
pNK4404
https://benchling.com/s/seq-nl9lN0MCpySO3kOThj3P?m=slm-s5Y3vHxsDwaH7jYge0gv</td></tr>
<tr><td>nnH3H v2</td><td>bent
pNK116
https://benchling.com/s/seq-TVjqbAUDpSLtXwiAPO3g?m=slm-ExzXht21ELkHozzykx8B</td><td>hum
pNK189
https://benchling.com/s/seq-L4r5kVGHb1f7hBXDCThE?m=slm-pMFOFMzgm6ge3876qxah</td><td>ppas
pNK4169
https://benchling.com/s/seq-aRCkab6wDjYyXaRkdFYw?m=slm-zlyvnieVKI8rR9MrHo3v</td></tr>
<tr><td>nnLuz WT</td><td>bent
pX018
https://benchling.com/s/seq-OJbc805At80x2L7dquDN?m=slm-FxbJkfNO6T8ibDTQvi4U

Plasmid from the Ref [1]</td><td colspan="2">ppas/hum
pNK5756
https://benchling.com/s/seq-VtvFVR5UJ7K1KfrVM2qb?m=slm-5hjDOc0iqHqSRDiPzWJl</td></tr>
<tr><td>nnLuz v4</td><td>bent
pNK115
https://benchling.com/s/seq-y3dUN4bGeogUKptVuOyK?m=slm-KBkqCwCQ64I3uPpx8C6a</td><td colspan="2">ppas/hum
pNK845
https://benchling.com/s/seq-qMJVqAQHeop5RyZR3hia?m=slm-ew9YhTknPhMrPNEUlCOf</td></tr>
</table>

Supplementary Table 2. Plasmids for expression in mammalian cells.

ID	Plasmid description	Plasmid map
pNK5780	DVK_AF | pCMV - nnLuz WT ppas/hum - tSV40	https://benchling.com/s/seq-9tRkFeQVsuQn2Pv3DrF4?m=slm-8KKhqeqjiF99jNeFc0Mx
pNK5664	DVK_AF | pCMV - nnLuz v4 ppas/hum - tSV40	https://benchling.com/s/seq-szpjjEFox2RoBAzbCX12?m=slm-ihCzRTcKpFU1ZiX3PXHU
pNK294	DVK_AF | pCMV - nnH3H WT hum - tSV40	https://benchling.com/s/seq-DzJM6LiezPSHLR66y0Oa?m=slm-nifL6L9bWmLeokcWZxID
pNK291	DVK_AF | pCMV - nnH3H v2 hum - tSV40	https://benchling.com/s/seq-XBaFaZSOwkvtLAYwv2xb?m=slm-YQ3g3Fqr27hzRjO305Jl
pX136 Plasmid from the Ref [1]	C-vector | pCMV - CPH - tSV40	https://benchling.com/s/seq-TsINsVoYeWAlr0I8LE2B?m=slm-IK2nwHwYl62YHGfn66OL
pX137 Plasmid from the Ref [1]	C-vector | pCMV - npgA - tSV40	https://benchling.com/s/seq-scdZF7tmIp3uwCvN5cq5?m=slm-Kks6Aspwz3PoqvqcsPy
pNK6006	DVK_AF | pCMV - nnHispS hum - tSV40	https://benchling.com/s/seq-VFZ8LRO1nGvooM2ZpFnU?m=slm-i5txnmCnrxwnLcituwEQ
pNK3203	DVK_AF | pCMV - mcitHispS - tSV40	https://benchling.com/s/seq-Sl4CaS08bfKDONntgU7w?m=slm-2ClaPJMFeqOFmn17jpEK
pNK3205	DVK_AF | pCMV - aoHispS - tSV40	https://benchling.com/s/seq-GrSF5SUbkagxp2IlhnPQ?m=slm-6LBWbRoQOW6JxSoFb60O
pNK3209	DVK_AF | pCMV - gnHispS - tSV40	https://benchling.com/s/seq-4x5o1iTEz6fzVaNglOPp?m=slm-RW4RC1lOF1cCNI620uWn
pNK3210	DVK_AF | pCMV - agHispS - tSV40	https://benchling.com/s/seq-iVe4IVD0plINdsP9vqHD?m=slm-OrPPzVKHs4hh8xOpz3lV
pNK3061	DVK_AF | pCMV - amHispS - tSV40	https://benchling.com/s/seq-Thpiq3qd8mE6wqF8S9IH?m=slm-7d4kjn7cQpAcxWxnZJKf
pNK6009	DVK_AF | pCMV - afHispS - tSV40	https://benchling.com/s/seq-46AvNq6LGOdOUlDBUN3w?m=slm-UXc1lNm2SzBdDm7kOkqR
pNK6265	DVK_AF | pCMV - FFLuc - tSV40	https://benchling.com/s/seq-7Xlv3YGxuGpSS2LwOtL1?m=slm-BAthmBp9g0mgHBNRXE9U
pNK6272	DVK_AF | pCMV - nanoLuc - tSV40	https://benchling.com/s/seq-hKIxkDzoUtyCV2BNSFGG?m=slm-ak5BeGANecNrrh2YBCMy
pNK6593	pCIDAR | pCMV - co-iLuxA - tSV40	https://benchling.com/s/seq-gv6bAgIxSUKo7oL6fSpC?m=slm-Qzm79rNdEhJXqfIS0wdA
pNK6595	pCIDAR | pCMV - co-iLuxB - tSV40	https://benchling.com/s/seq-HMiGjWoHkFY8FBW98euO?m=slm-2FSrSwfQto2PRzHxLjlf
pNK6599	pCIDAR | pCMV - co-iLuxC - tSV40	https://benchling.com/s/seq-Qpxe6tYz0yodSVODQxV2?m=slm-OKpcxkjebRkuwjvO9WMK
pNK6612	pCIDAR | pCMV - co-iLuxD - tSV40	https://benchling.com/s/seq-iqTwv6g9sSTn6rNCfPlh?m=slm-eEGfZCyUnLIjqxgWe89l
pNK6604	pCIDAR | pCMV - co-iLuxE - tSV40	https://benchling.com/s/seq-CLqFGnVM6M6SmYjN3UUG?m=slm-JSqi8xAqU8BNcmVf3S1i

pNK6614	pCIDAR | pCMV - frp - tSV40	https://benchling.com/s/seq-R23jcci4BI8jYpD6GdTX?m=slm-FMb8LI5vgoUsUxeBiI2t

Supplementary Table 3. Plasmids for expression in *P. pastoris*.

ID	Plasmid description	Plasmid map
pNK1508	pGAP-Hyg | NpgA	https://benchling.com/s/seq-wSZV0ADJQVfKbmbkxl0Y?m=slm-sq6RcovW5iO0rsL5RbWt
pNK5869	pGAP-Z | nnHispS	https://benchling.com/s/seq-8YnNqUjSobFUjkreIKNu?m=slm-RUJJDkJlV0g1aTaV5D9m
pNK3293	pGAP-Z | afHispS	https://benchling.com/s/seq-4jfheFWnNITRMy1PwWTP?m=slm-dyPZ8ct3kU3f2Fezqz4C
pNK5871	pGAP-Z | agHispS	https://benchling.com/s/seq-O8n0A9ZKqB7yHbIlNZef?m=slm-oz7B27p56mnio4h6l3op
pNK5889	pGAP-Z | amHispS	https://benchling.com/s/seq-sfMSGdiOiVMc2ryRTMaw?m=slm-czE8bLlmIHfqO3xlGN1j
pNK5913	pGAP-Z | aoHispS	https://benchling.com/s/seq-thvcdYtpRkUrBoZue81q?m=slm-TBlqYJmPQSvJONDXGVuU
pNK3292	pGAP-Z | gnHispS	https://benchling.com/s/seq-u8RO6pOdToKTUezy0tNp?m=slm-rzF6IQODcZhEocnRIfin
pNK3287	pGAP-Z | mchHispS	https://benchling.com/s/seq-2Rum32em3qlIcnp2fHOA?m=slm-vN5VBNmZLjkLobrLTRjE
pNK5867	pGAP-Z | mcitHispS	https://benchling.com/s/seq-IVcfX0nBxssWmeqxVt4p?m=slm-xcpaq3j8GRPGqL4HeaPq
pNK3222	pGAP-Z | ngHispS	https://benchling.com/s/seq-MTitklOHQIMVTPaDl0J2?m=slm-jhVXTYbOYKaiAulldHK4
pNK3017	pGAP-Z | psHispS #1	https://benchling.com/s/seq-TXzLZk0INjCNEIKCzDFw?m=slm-appoLNampXAj4zTUW0Xn
pNK3019	pGAP-Z | psHispS #2	https://benchling.com/s/seq-F4vpXaWNjX1JIVko5gf7?m=slm-OpJMfSwnxB4zgdPGuB66
pNK5709	pGAP-Hyg | nnH3H WT	https://benchling.com/s/seq-O1IxHpymMnUomzI3TZ4E?m=slm-tn4sNABx3A5f0dhO1DFg
pNK5712	pGAP-Hyg | nnH3H v2	https://benchling.com/s/seq-7tWRDMIL0skZFAMTJpEW?m=slm-1R8J6U1iC3NIMvnzk5lu
pNK5788	pGAP-Kan | nnLuz WT	https://benchling.com/s/seq-7CxEVTT20bRKNS3m6yM8?m=slm-61XdsunIkiFEZRFMTNEo
pNK5785	pGAP-Kan | nnLuz v4	https://benchling.com/s/seq-UuMufCjCBXGcjWrr2lG2?m=slm-OW3bR80rxCmEdPJaoJr9
pNK6624	pGAP-Hyg | nnLuz WT - HiBiT	https://benchling.com/s/seq-Amwp8L7rUC8h5RuCcu6V?m=slm-l8TtpZQt5p5SJN1Zpxbf
pNK996	pGAP-Hyg | nnLuz v4 - HiBiT	https://benchling.com/s/seq-HXE8ZVTGkGFG8I0ci6Uc?m=slm-8lTOVDp1czXYE4v8tg7N

Supplementary Table 4. Plasmids for expression in plants.

ID	Plasmid description	Plasmid map
pN094	Level 1 | p35s - NpgA - tOCS	https://benchling.com/s/seq-o1FM7PTx7f1izd0G5Zte?m=slm-Jtuehn5Ny7J7hoaUjYQZ
pNK077	Level 1 | pCmYLCV - NpgA - tATP	https://benchling.com/s/seq-Pru2MS278Nrr7yxjNQBK?m=slm-txwiWQz5LC3quw08eTel
pX028	Level 1 | p35s - nnHispS - tOCS	https://benchling.com/s/seq-PjqSpOXySuN3KAXDS4dp?m=slm-4KWYBc2Yib5mOyflX6z6
pNK2714	Level 1 | p35s - mcitHispS - tOCS	https://benchling.com/s/seq-OTdGulhG7uBSySd06gMy?m=slm-5GhfWLpDsw4YcJ5tad2D
pNK2748	Level 1 | p35s - gnHispS - tOCS	https://benchling.com/s/seq-NxvVSys9zCAFoIkKhApg?m=slm-PB4MJ2q1q1oaChmNrnj4
pNK5739	Level 1 | p35s - afHispS - tOCS	https://benchling.com/s/seq-co3aAC8InccrH6PLg6ao?m=slm-YjFuVC00COdBatCFGoq8
pNK2674	Level 1 | p35s - aoHispS - tOCS	https://benchling.com/s/seq-4QYpwefWZvmOhWBtxGya?m=slm-axGMB89CJrSpn9pO8NP5
pNK2711	Level 1 | p35s - agHispS - tOCS	https://benchling.com/s/seq-FHSP8wG3ImkLj1fbh3SI?m=slm-5wOHQUJA3FHzdkrL8btD
pNK2816	Level 1 | p35s - amHispS - tOCS	https://benchling.com/s/seq-6wlIrkjhfRYO4wkBfDVU?m=slm-hQWhMPCL5O0AjtHHjeWV
pNK2715	Level 1 | p35s - psHispS #1 - tOCS	https://benchling.com/s/seq-8GEGPbckUqbldANTIVhQ?m=slm-vzz7g32kmx8Nbs5xnqO6
pNK2716	Level 1 | p35s - psHispS #2 - tOCS	https://benchling.com/s/seq-KzI5qzQPg8jkd8edxE7J?m=slm-hYP8T6y9mL8SHIDBE2gC
pNK2857	Level 1 | p35s - ngHispS - tOCS	https://benchling.com/s/seq-CaQQIlPJ46K8FZ0l0Fwd?m=slm-HhAV8IhpaKtZiFjjiE02
pNK2676	Level 1 | p35s - mchlHispS - tOCS	https://benchling.com/s/seq-PEYW7vARjkcnANQjjOo6?m=slm-W1J3vqyaufS5ycDGgBNL
pNK093	Level 1 | pFMV - nnH3H WT - tNOS	https://benchling.com/s/seq-DnBkfjWoak29sgieFLoF?m=slm-U5OjKtgT09Z9dMdIClrw
pNK156	Level 1 | pFMV - nnH3H v2 - tNOS	https://benchling.com/s/seq-j5jjkkjuRK5VNLsbXtGp?m=slm-aFvsHms19B6JefbsmpoG
pNK5668	Level 1 | p35s - nnLuz WT - tAct2	https://benchling.com/s/seq-jkgdQelPsEaWW9MUfwc2?m=slm-6Ili6zm0m4eWaNoON453
pNK155	Level 1 | p35s - nnLuz v4 - tAct2	https://benchling.com/s/seq-JHhqKcFYX05LiRKLLTaa?m=slm-YYutXmSCiqovJHglaSB1
pX030	Level 1 | p35s - nnCPH - tOCS	https://benchling.com/s/seq-7XBDGX8yci30y36InhGw?m=slm-76s6l2kobMN6e46mKQkd
pNK6260	Level 1 | p35s(0.4 kb) - FFLuc - tOCS	https://benchling.com/s/seq-rLXcKcvdkt8DpM9o7Xcq?m=slm-oEP8R0c7iWC65YScfAlF
pNK6269	Level 1 | p35s(0.4 kb) - nanoLuc - tOCS	https://benchling.com/s/seq-BKRLDkmTAUWRNmfBmHUj?m=slm-SCGfQZMUgjcIlOxp4sC4
pNK6357	Level 1 | p35s(0.4 kb) - iLuxA - tOCS	https://benchling.com/s/seq-5Whqyp9xxIKYWcy7IK4X?m=slm-Mgowq7sYFAWAZguGjMon

<table>
<tr><td>pNK6359</td><td>Level 1 | p35s(0.4 kb) - iLuxB - tOCS</td><td>https://benchling.com/s/seq-jGulTHkqmFwT15pJwMr6?m=slm-uL9SwDh90Cl2zzK95cdS</td></tr>
<tr><td>pNK6361</td><td>Level 1 | p35s(0.4 kb) - iLuxC - tOCS</td><td>https://benchling.com/s/seq-HhJczSCQXbtrDqscfakv?m=slm-354btwib8r1hMXylnzVj</td></tr>
<tr><td>pNK6363</td><td>Level 1 | p35s(0.4 kb) - iLuxD - tOCS</td><td>https://benchling.com/s/seq-X5tMVTn10Ll0yHXxXnzX?m=slm-TLciHUgakLS3DGq0FdIL</td></tr>
<tr><td>pNK6365</td><td>Level 1 | p35s(0.4 kb) - iLuxE - tOCS</td><td>https://benchling.com/s/seq-04VdDNUXuThYsrm9J5zk?m=slm-nSOsz93zbbVpFWFwy9Ql</td></tr>
<tr><td>pNK6367</td><td>Level 1 | p35s(0.4 kb) - frp - tOCS</td><td>https://benchling.com/s/seq-Zc7BGRR0pssIBkAvocrn?m=slm-pRsZjLtm8waki3ukLPjo</td></tr>
<tr><td>pNK6369</td><td>Level 1 | p35s(0.4 kb) -chloroplast_transit_peptide-iLuxA - tOCS</td><td>https://benchling.com/s/seq-xKBn7UOj4YrLkSVk0eYK?m=slm-bVS3mBcnBoO3Vuzln1IF</td></tr>
<tr><td>pNK6371</td><td>Level 1 | p35s(0.4 kb) -chloroplast_transit_peptide-iLuxB - tOCS</td><td>https://benchling.com/s/seq-rZYCXpgDbgnwUQRAuxI2?m=slm-I5857JDdPVz95vCGJM1o</td></tr>
<tr><td>pNK6373</td><td>Level 1 | p35s(0.4 kb) -chloroplast_transit_peptide-iLuxC - tOCS</td><td>https://benchling.com/s/seq-rwCYI6OSeCBI5kMeSfkq?m=slm-fncQqex2ookrBWzOpVI8</td></tr>
<tr><td>pNK6375</td><td>Level 1 | p35s(0.4 kb) -chloroplast_transit_peptide-iLuxD - tOCS</td><td>https://benchling.com/s/seq-oDmtu6doZzDV1U1asoRB?m=slm-IyDksiP3zGwNiLuVap2j</td></tr>
<tr><td>pNK6377</td><td>Level 1 | p35s(0.4 kb) -chloroplast_transit_peptide-iLuxE - tOCS</td><td>https://benchling.com/s/seq-u1D4fbNnAqPQrlBKY1w6?m=slm-Xh3JTMwHXJcfEg55R1nb</td></tr>
<tr><td>pNK6379</td><td>Level 1 | p35s(0.4 kb) --chloroplast_transit_peptide-frp - tOCS</td><td>https://benchling.com/s/seq-U65pjnwhXL2EjjZf3I1L?m=slm-WC7YkQy3Sij4YSH7ox7u</td></tr>
<tr><td>pNK511</td><td>nnHispS, nnH3H_v2, nnLuz_v4, nnCPH
Level P | pNOS - KanR - tOCS | p35s - nnHispS - tOCS | p35s - Luz v4 - tAct2 | p35s - nnCPH - tOCS | pFMV - nnH3H v2 - tNOS</td><td>https://benchling.com/s/seq-GTuIg6Rx7L3ISXacqiIg?m=slm-h322lbmf0Y36Q9rbSwyA</td></tr>
<tr><td>pNK497</td><td>FBP2
Level P | pNOS - KanR - tOCS | p35s - nnHispS - tOCS | pCmYLCV - NpgA - tATP | p35s - Luz v4 - tAct2 | p35s - nnCPH - tOCS | pFMV - nnH3H v2 - tNOS</td><td>https://benchling.com/s/seq-DqozAu4sYn937p5UdD6Z?m=slm-m38crKINPSj2Sg8dkAjQ</td></tr>
<tr><td>pNK3071</td><td>FBP3
Level P | pNOS - KanR - tOCS | p35s - mcitHispS - tOCS | pCmYLCV - NpgA - tATP | p35s - Luz v4 - tAct2 | p35s - nnCPH - tOCS | pFMV - nnH3H v2 - tNOS</td><td>https://benchling.com/s/seq-NNKqOeRGCYTISxRYy3SQ?m=slm-78WwJlaPG6mU0O6gRBCC</td></tr>
<tr><td>pNK6537</td><td>mcitHispS, nnH3H_v2, nnLuz_v4, nnCPH, NpgA
Level P | pNos - KanR - tOCS | p35s - mcitHispS - tOCS | pFMV - nnH3H_v2 - tNOS | p35s - nnLuz_v4 - tAct2 | p35s- nnCPH - ocsT | pCmYLCV - npgA - tATP</td><td>https://benchling.com/s/seq-ihOTINEWjRUG85MDv5Ng?m=slm-npKsDdUw7TzPk38MbvYV</td></tr>
<tr><td>pNK6541</td><td>nnHispS, nnH3H_v2, nnLuz_v4, nnCPH, NpgA
Level P | pNos - KanR - tOCS | p35s - nnHispS - tOCS | pFMV - nnH3H_v2 - tNOS | p35s - nnLuz_v4 - tAct2 | p35s- nnCPH - ocsT | pCmYLCV - npgA - tATP</td><td>https://benchling.com/s/seq-I5IRAnDAKaZtcokLeGbe?m=slm-OJwidfOKcolTkzZFRuqJ</td></tr>
</table>

Supplementary Table 5. Transgenic plant lines used in this study.

Plant species	Plasmid, pathway	Line ID
Arabidopsis thaliana	pNK497, FBP2	AT6121
		AT6123
	pNK3071, FBP3	AT6124
		AT6125
		AT6126
Chrysanthemum morifolium	pNK497, FBP2	CHR2105
Nicotiana benthamiana	pX037, FBP1	NB021 from ref[1]
	pNK511	NB097
		NB253
		NB1291
	pNK497, FBP2	NB072
		NB081
		NB103
		NB104
	pNK3071, FBP3	NB2186
		NB5730
		NB5732
		NB5733
		NB5734
Nicotiana tabacum	pX037, FBP1	NT001 from ref[1]
	pNK497, FBP2	NT2641
		NT2817
		NT2819
Petunia hybrida	pNK497, FBP2	PET373
	pNK3071, FBP3	PET2720
		PET2234
Populus canadensis	pX037, FBP1	POP5
	pNK497, FBP2	POP11

Supplementary information references

1. Mitiouchkina, T. *et al.* Plants with genetically encoded autoluminescence. *Nat. Biotechnol.* (2020) doi:10.1038/s41587-020-0500-9.
